# Shaping brain structure: Genetic and phylogenetic axes of macroscale organization of cortical thickness

**DOI:** 10.1126/sciadv.abb3417

**Published:** 2020-09-25

**Authors:** Sofie L. Valk, Ting Xu, Daniel S. Margulies, Shahrzad Kharabian Masouleh, Casey Paquola, Alexandros Goulas, Peter Kochunov, Jonathan Smallwood, B. T. Thomas Yeo, Boris C. Bernhardt, Simon B. Eickhoff

**Affiliations:** 1 Institute of Neuroscience and Medicine (INM-7: Brain and Behavior), Research Centre Jülich, Jülich, Germany.; 2 Institute of Systems Neuroscience, Heinrich Heine University Düsseldorf, Düsseldorf, Germany.; 3 Otto Hahn Group Cognitive Neurogenetics, Max Planck Institute for Human Cognitive and Brain Sciences, Leipzig, Germany.; 4 Center for the Developing Brain, Child Mind Institute, New York, NY, USA.; 5 Frontlab, Centre National de la Recherche Scientifique Institut du Cerveau et de la Moelle Épinière, Paris, France.; 6 McConnell Brain Imaging Centre, Montreal Neurological Institute and Hospital, McGill University, Montreal, QC, Canada.; 7 Institute of Computational Neuroscience, University Medical Center Hamburg-Eppendorf, Hamburg University, Hamburg, Germany.; 8 Maryland Psychiatric Research Center, University of Maryland School of Medicine, Baltimore, MD, USA.; 9 York Neuroimaging Center, University of York, York, UK.; 10 Department of Electrical and Computer Engineering, Centre for Sleep and Cognition, Centre for Translational MR Research and N.1 Institute for Health, National University of Singapore, Singapore, Singapore.; 11 Athinoula A. Martinos Center for Biomedical Imaging, Massachusetts General Hospital, Charlestown, MA, USA.; 12 NUS Graduate School for Integrative Sciences and Engineering, National University of Singapore, Singapore, Singapore.

## Abstract

The topology of the cerebral cortex has been proposed to provide an important source of constraint for the organization of cognition. In a sample of twins (*n* = 1113), we determined structural covariance of thickness to be organized along both a posterior-to-anterior and an inferior-to-superior axis. Both organizational axes were present when investigating the genetic correlation of cortical thickness, suggesting a strong genetic component in humans, and had a comparable organization in macaques, demonstrating they are phylogenetically conserved in primates. In both species, the inferior-superior dimension of cortical organization aligned with the predictions of dual-origin theory, and in humans, we found that the posterior-to-anterior axis related to a functional topography describing a continuum of functions from basic processes involved in perception and action to more abstract features of human cognition. Together, our study provides important insights into how functional and evolutionary patterns converge at the level of macroscale cortical structural organization.

## Teaser

Functional and evolutionary patterning converges at the level of macroscale cortical structural organization.

## INTRODUCTION

A fundamental question in neuroscience is how the structure of the cortex constrains its function. Over the course of almost a century, numerous studies have shown that the cerebral cortex is organized along dimensions that reflect systematic variations in features of brain structure and function such as laminar differentiation, gene expression, and structural and functional connectivity (*1*). These dimensions have been suggested to reflect the timing of neurogenesis (*2*–*4*). A potential mechanism for the source of neurogenetic differentiation of brain regions is described by the dual origin theory (*3*, *4*). This theory conceptualizes cortical areas as emerging from waves of laminar differentiation that spring from the piriform cortex (paleocortex) and the hippocampus (archicortex). The dual structure might be rooted in heterochronous ontological axes in the developing cortex (*3*, *4*). It has been suggested that a sequential progression of cortical architectonic differentiation arises from each of both origins, which leads to the six-layered proisocortex and isocortex, resulting in a sensory-fugal organization (*5*).

The systematic topological organization of the cerebral cortex has been proposed to reflect an architecture that optimizes the balance of externally and internally oriented functioning, which is critical for flexible human cognition (*6*). For example, association cortex is located at maximal distance from regions of primary cortex that are functionally specialized for perceiving and acting in the here and now. This increased spatial distance from primary cortex may allow association cortex to take on functions that are only loosely constrained by the immediate environment, allowing internal representations to contribute to cognition and so enhancing the flexibility and evolutionary fitness of behavior (*6*, *7*). Accordingly, understanding how the structure of the cortex scaffolds function in a flexible manner requires understanding how macroscale structural features of the organization of the human cortex emerge. Moreover, previous work has implicated macroscale organizational axes of structure and function in the impact and progression of pathology. For example, Parkinson’s and Alzheimer’s disease have been proposed to follow a trajectory, in which underlying anatomical axes determine the sequence in which specific regions and networks are progressively affected at different disease stages (*8*). Recently, we have been able to show that functional abnormalities in autism spectrum disorder relate to systematic disruptions in large-scale functional organization, providing a parsimonious reference frame in which the heterogeneous symptoms of autism spectrum disorder can be understood (*9*).

Although the importance of macroscale axes of cortical organization in cognition and pathology are now recognized, the degree to which these topological features of the cerebral cortex are genetically determined remains incompletely understood. Cortical thickness is a widely used morphological measure and has been related to the neuronal density, cytoarchitecture, and structural hierarchical organization of the cerebral cortex (*10*, *11*). Measured across a population, local brain structure shows marked patterns of covariation across the cerebral cortex, termed “structural covariance.” These macroscale patterns in cortical thickness provide a model of shared maturational and genetic effects on morphology (*12*–*16*) and have been linked to both structural and functional connectivity (*17*, *18*). Structural covariance has been reported to be organized in various local, genetically determined, communities (*12*–*14*). Recent work shows that interregional genetic correlation is determined by two organizational principles: (i) regions are strongly genetically correlated with their counterparts in the opposite cerebral hemisphere, and (ii) regions are highly genetically correlated with geometrically nearby regions (*13*). The local processes that govern the observed distribution of cortical thickness are reasonably well understood. For example, associations with structural and functional connectivity may arise due to shared trophic changes at the synaptic and cellular levels (*19*) and/or reflect coupled expression of genes enriched in supragranular layers (*20*) that are associated with transcriptomic similarity of local brain regions (*21*). Both of these effects converge with postmortem interregional correlations of gene expression (*22*). Developmentally, macroscale patterns of cortical thickness mature with age, possibly because of synchronized neurodevelopment (*15*) and the expression of common genetic cues during early cortical development (*15*).

Together, contemporary theory suggests that (i) macroscale patterns in cortical organization make an important contribution to human cognition and (ii) cortical thickness covariance reflects shared maturational and genetic processes and is organized in multiple communities. However, the reason for their particular spatial relationship remains unclear. Our current study sought to directly examine the genetic origin of spatial organization of macroscale cortical features. We used unsupervised learning methods to construct large-scale organizational gradients that underpin the structural covariance across the cortex. In contrast to clustering-based decompositions of the brain into discrete communities (*23*), cortex-wide gradient mapping techniques describe neural structure and function in a low-dimensional space, or coordinate system, that reflects the macroscale patterns that underpin the observed neural data. We used this approach to describe the structural covariance in humans and in nonhuman primates and to evaluate whether these dimensions of variation are genetically determined. In particular, we used a twin-design on the basis of the Human Connectome Young Adult sample (S1200) using Sequential Oligogenic Linkage Analysis Routines (SOLAR; www.solar-eclipse-genetics.org; Solar Eclipse 8.4.0.) to evaluate genetic correlation of local cortical thickness across the cortical mantle. In a second analysis, we evaluated the phylogenetic basis of macroscale patterns of structural covariance by comparing the large-scale gradients in macaque monkeys [PRIMatE Data Exchange (PRIME-DE)] (*24*) with those seen in humans. Last, we compared the axes of macroscale organization of cortical thickness in humans and macaques with organizational axes expected based on the theory of dual origin (*3*).

Foreshadowing our results, both analyses supported that the two main organizational patterns that describe macroscale patterns of cortical thickness were driven by genetic factors. Pedigree models revealed that macroscale patterns of cortical thickness covariance were highly influenced by genetics, especially in prefrontal cortex, highlighting the role of genetics in shaping brain structure in regions functionally associated with complex features of human cognition. We also observed a similar macroscale organization in humans and macaques, suggesting that these axes are phylogenetically conserved in primates. Moreover, we found an inverse relationship between archicortex (hippocampus) and paleocortex (olfactory cortex) distance and the inferior-to-superior organization gradient in humans and macaques, aligning covariance topology with the dual origin theory. Together, these analyses highlight the important role that genetic processes play in determining the large-scale organization of cortical structure and so provide an important window into the innate architecture supporting human cognition.

## RESULTS

### Axes underlying macroscale coordination of cortical thickness

**Fig. 1:**
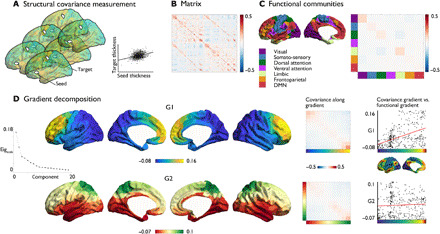
Large-scale organization of structural covariance. (**A**) Measuring structural covariance of thickness. (**B**) Structural covariance matrix using 400 Schaefer parcels (*25*). (**C**) Mean correlation within functional network community (*23*). DMN, default mode network. (**D**) Gradient decomposition. Left: Principal (G1) and second (G2) macroscale gradient of structural covariance. Middle: Structural covariance strength within and between gradient bins (10 bins). Right: Correlation of G1 and G2 with the principal gradient of resting-state fMRI (*28*).

We started our analysis by evaluating the topological organization of interregional cortical thickness correlations [henceforth referred to as structural covariance (Fig. 1)]. We used the mean thickness within 400 parcels (*25*) to create group-level covariance maps based on individual thickness values of participants from the Human Connectome Project (HCP; S1200). When computing the macroscale organization of cortical thickness, we controlled for the effects of age, sex, and global thickness. First, we evaluated the average structural covariance as a function of brain network organization (*23*). Within-community covariance (mean ± SD, 0.05 ± 0.03) was higher than between-community covariance (mean ± SD, −0.01 ± 0.02), *P* < 0.001, and visual, sensory-motor, fronto-parietal, and default mode networks had higher covariance within network relative to random networks [false discovery rate (FDR) *q* < 0.05], in line with previous work (Fig. 1B) (*26*).

We then implemented diffusion map embedding; a method previously used in functional connectivity and microstructural profile covariance networks (*27*, *28*). Diffusion map embedding allows local and long-distance connections to be projected into a common space (*28*). The resulting components are unitless and identify the position of nodes along axes that describe the dominant differences in a given nodes’ connectivity profile. The principal gradient (G1) in structural covariance followed a posterior-anterior trajectory from occipital regions to the frontal cortex and accounted for 17% of the variance in the thickness covariance data. Next, we examined the covariance values as a function of the structural gradient. We divided the structural gradient into 10 equally sized bins and plotted the average covariance values across bins. We observed that covariance was highest within bins and negative between both apexes of the principal gradient. Gradient bins within the frontal cortex showed heightened covariance between each other (Fig. 1C). Next, we evaluated the association between the posterior-anterior gradient and a previously reported large-scale gradient of functional connectivity, reflecting functional hierarchy (*28*). The functional gradient showed a positive correlation with the posterior-anterior structural covariance gradient G1 (*r* = 0.47, *P*_spin < 0.005) using non-parametric spin tests to account for spatial autocorrelation (Fig. 1) (*29*).

The second gradient (G2) followed an inferior-superior pattern with end points in superior parietal lobe and lingual gyrus, respectively, and explained 13% of the variance. Binning G2, we observed highest covariance within and between superior regions, negative covariance between both gradient end points, and a positive diagonal indicating covariance patterns where highest within and not between bins. The inferior-superior covariance gradient did not show a relation with the principal functional gradient (*r* = 0.05, *P* > 0.1) (Fig. 1).

Findings were reproducible in a different dataset [eNKI (enhanced NKI-Rockland sample), *n* = 799, age 8 to 85 years] using different preprocessing pipelines of thickness (CIVET and FreeSurfer 6.0), and parcellation methods [Desikan-Killiany (*30*), Glasser atlas (*31*), and Schaefer atlas (*25*) (800 parcels); fig. S1 and Supplementary Results], and gradients were robust to variations in kernel, approach, and cutoff value (fig. S2). The principal and second gradients, as well as gradients 3 and 4, showed comparable patterning bilaterally, while gradients 5 to 8 showed lateralization effects (fig. S3 and Supplementary Results). Follow-up analysis indicated that the gradients of macroscale organization of cortical thickness existed above and beyond geodesic distance constraints and aligned with previously reported gradients in cortical microstructure (T1w/T2w) (*27*) (Supplementary Results). Conducting a meta-analytical functional decoding analysis using the Neurosynth database, we observed marked variation of function along both macroscale organizational gradients of thickness (Supplementary Results).

### Macroscale organization of cortical thickness is genetically determined

**Fig. 2:**
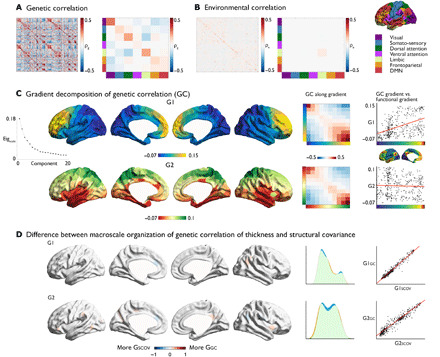
Large-scale organization of genetic correlation of cortical thickness. (**A**) Left: Genetic correlation of local cortical thickness in the Schaefer parcellation (*25*). Right: Mean genetic correlation within/between functional communities (*23*). (**B**) Left: Environmental correlation of cortical thickness. Middle: Mean environmental correlation within/between functional communities (*23*). Right: Functional communities (*23*). (**C**) Gradient decomposition. Left: Principal (G1) and second (G2) macroscale gradient. Middle: Average genetic correlation within and between gradient bins (10 bins). Right: Correlation between G1 and G2 and principal gradient in resting-state fMRI (*28*). (**D**) Left: Parcel-wise difference between the structural covariance gradients (G_SCOV_) and the genetic correlation gradients (G_GC_). Blue indicates higher gradient ranking in G_SCOV_, and red indicates higher gradient ranking in G_GC_. Middle: Density plots (blue, G_SCOV_; green is overlap; red, G_GC_) and Right: Scatter of the structural covariance and genetic correlation gradients.

Genetic correlation is based on the decomposition of structural covariance into genetic and environmental factors using the genetic similarity between individuals to estimate shared additive genetic effects. Using the heritability and genetic correlation of each parcel pair, we computed bivariate heritability to assess the proportion of phenotypic correlation explained by genetic factors. Mean heritability (*h*^2^) of thickness was (mean ± SD) 0.28 ± 0.11 (table S2). Overall, there was a very high correspondence between genetic correlation and bivariate heritability (*r* = 0.95) and 75.4 ± 8% (mean ± SD) of the phenotypic correlation could be attributed to genetic factors (fig. S4). Moreover, we observed high correlation between thickness covariance and genetic correlation of thickness (*r* = 0.67) and environmental correlation of thickness (*r* = 0.41) across all nodes (fig. S4). Patterns of genetic correlation were highest within (mean ± SD, 0.14 ± 0.08), rather than between (mean ± SD, −0.02 ± 0.05), functional communities, *P* < 0.001, and visual, dorsal attention, fronto-parietal, and default mode network had higher genetic correlation within network relative to random networks at FDR *q* < 0.05 (Fig. 2A). Although much lower than genetic correlations, environmental correlations were also stronger within functional network (within: mean ± SD, 0.01 ± 0.02; between: 0.00 ± 0.01; *P* < 0.001; Fig. 2B).

Performing whole-brain gradient decomposition on the genetic correlation maps, we observed almost identical large-scale gradients as in the structural covariance (structural covariance G1 versus genetic correlation G1: *r* = 0.98; structural covariance G2 versus genetic correlation G2: *r* = 0.96). The principal genetic gradient explained 18% of the variance, traversing a posterior-anterior axis. Binning the gradient, we observed (i) a diagonal axis of genetic correlation, indicating stronger association within, rather than between, gradient bins, and (ii) negative genetic correlation between both gradient apexes. Genetic correlation was strongest in the frontal cortex.

The second gradient explained 13% of the variance and reflected a similar inferior-superior axis as was seen in the structural covariance gradients. Genetic correlation was strongest between regions at similar levels of the inferior-superior gradient, with regions in both apexes showing stronger genetic correlations. Similar to our observations in structural covariance, we observed a positive relationship between G1 and the principal gradient of functional organization (*r* = 0.48, *P*_spin < 0.005) but not for G2 [*r* = 0.02, *P* = not significant (ns)]. Environmental correlations were organized along a posterior-anterior and inferior-superior axis as well, explaining 13 and 11% of the variance, respectively (fig. S5).

### Macroscale organization of cortical thickness in macaques

**Fig. 3:**
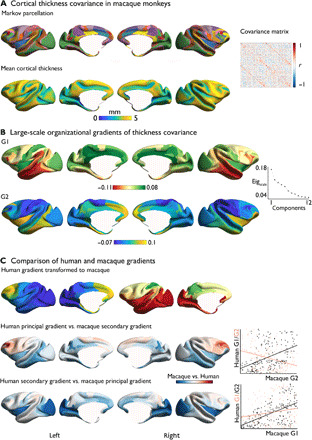
Structural covariance gradient in macaque monkeys. (**A**) Top: Markov parcellation (*32*). Bottom: Mean cortical thickness in 41 macaques from three independent sites (Davis, Oxford, and Newcastle). Right: Structural covariance of thickness matrix in macaques. (**B**) Gradient decomposition: primary gradient (G1) and secondary gradient (G2) of the structural covariance matrix. (**C**) Comparison of human and macaque gradients. Red indicates a higher gradient ranking in humans, whereas blue indicates a higher gradient ranking in macaques. Scatter plots indicate the association between human posterior-anterior covariance gradient (G1, black) and human inferior-superior covariance (G2, red) and macaque principal gradient (G1, top scatterplot) and secondary gradient (G2, bottom scatterplot).

Thus far, our analysis suggests that the macroscale organization of cortical structural covariance in humans shows high concordance among identical twins indicating a genetic influence. Our next analysis evaluated the genetic contribution to macroscale dimensions of cortical structure by examining its phylogenetic stability. To achieve this goal, we examined the topology of large-scale gradients in 41 macaque monkeys from the PRIME-DE (*24*). We created a structural covariance matrix on the basis of cortical thickness of 41 macaques using parcels based on the Markov atlas (*32*) and applied a similar analysis as for humans (see Materials and Methods). The principal and second gradient of the macaque monkey are presented in Fig. 3. Similar to the gradients of structural covariance in humans, we observed that the topological organization of macaque monkey’s structural covariance was also well described by both a posterior-anterior and inferior-superior component. In macaques, the ordering of the components was reversed with the inferior-superior gradient explained 17% of the variance, whereas the posterior-anterior gradient explained 13 % of the variance. The principal gradient stretched from inferior anterior temporal to sensory-motor cortex, and the secondary gradient stretched from sensory-motor to frontal cortex.

Last, using a recently-proposed cross species alignment (weighted functional alignment) (*33*), we transformed human gradients to macaque cortex and compared them with the gradients in macaques directly. We observed similar profiles for both the posterior-anterior gradient [*r* = 0.51, 95% confidence interval (CI) (0.39, 0.61), *P* < 0.0001] and inferior-superior gradient [*r* = 0.59, CI (0.47 0.69)] in humans and macaques. Similarities were stronger than between posterior-anterior gradient in humans and inferior-superior gradient in macaques [*r* = 0.10, CI [–0.03 0.23], *P* = ns] or inferior-superior gradient in humans and posterior-anterior gradient in macaques [*r* = –0.29, CI [–0.43 –0.15], *P* = 0.001].

### Macroscale organization of cortical thickness and the theory of dual origin

Last, we studied the genetic ontogeny of macroscale organization of cortical thickness in light of the dual origin theory of cortical development. This perspective assumes that cortical areas develop from waves of laminar differentiation that have their origin in either the piriform cortex (paleocortex) or the hippocampus (archicortex). The theory was established on histological investigations of the adult cortex of various reptiles and mammals (*3*). We evaluated the previously reported gradients in humans and macaques with respect to the geodesic distance from the paleocortex (olfactory cortex) and the archicortex (hippocampus) [similar to previous work (*34*)].

**Fig. 4:**
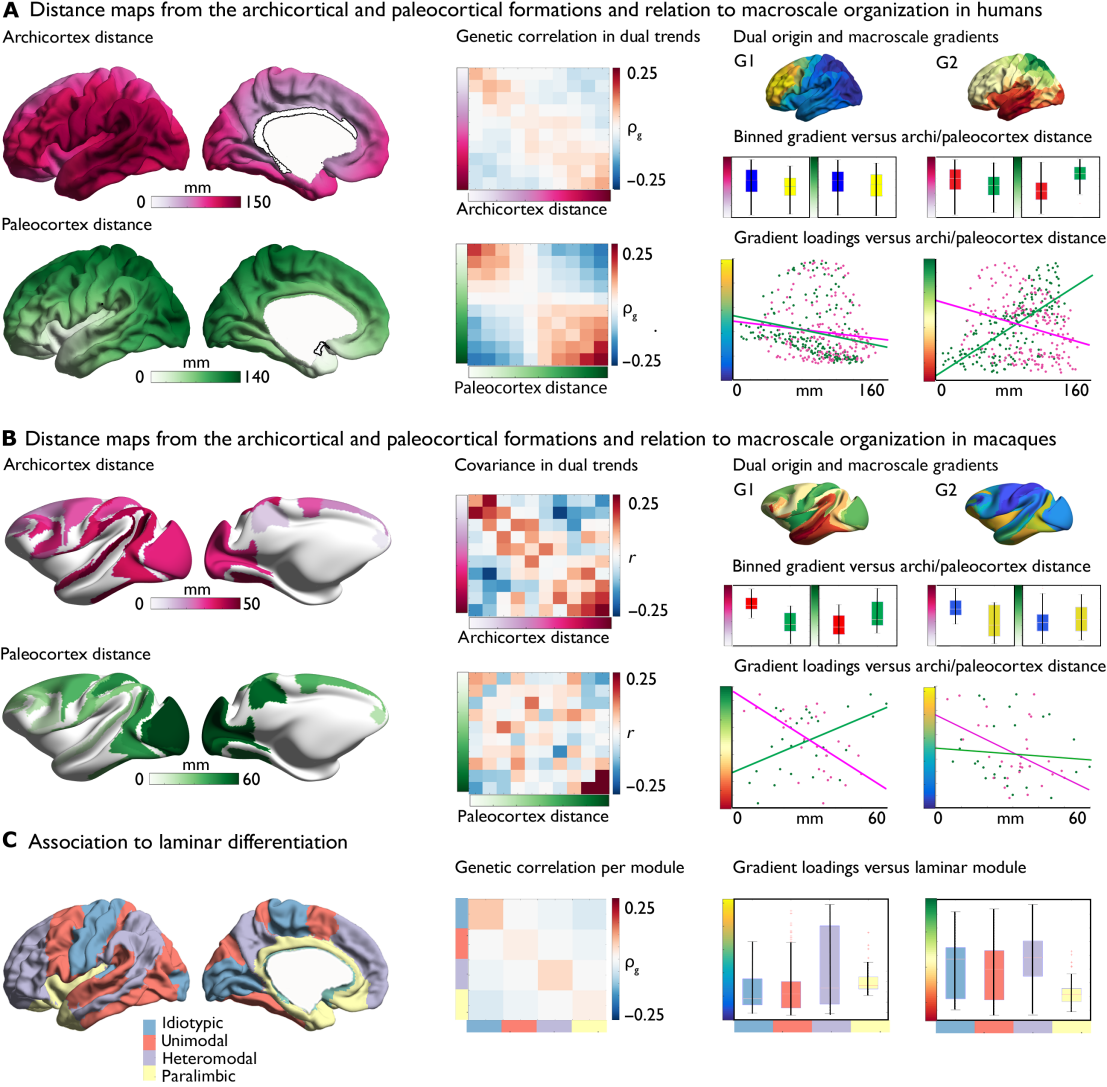
Cross-species topology of covariance as a function of the dual origin theory. (**A**) Left: distance from archicortex and paleocortex in humans. Middle: genetic correlation as a function of archi- and paleocortex distance (10 bins). Right: Association between G1 and G2 of genetic correlation of thickness and distance from archicortex and paleocortex in humans (both gradients binned in two bins and linear relationship between gradient and distance). (**B**) Left: Distance from archicortex and paleocortex in macaque monkeys (*34*). Middle: structural covariance as a function of archi- and paleocortex distance (10 bins) (*34*). Right: Association between G1 and G2 of thickness covariance and distance from archicortex and paleocortex in macaque monkeys (both gradients binned in two bins, as well as linear relationship between gradient and distance). (**C**) Left: Sensory-fugal maps of laminar differentiation (*77*). Middle: Genetic correlation as a function of laminar module. Right: Gradients versus laminal module.

We computed the geodesic distance from the archicortex and paleocortex in humans motivated by previous anatomical descriptions (Fig. 4A) (*3*) and evaluated its association to the principal and secondary gradient of genetic correlation of thickness (based on Fig. 2). We observed a dissociation between distance from paleocortex in inferior and superior proportions of the inferior-superior gradient by dividing the gradient in two [statistical energy test (*35*), *P* < 0.001]. We observed also a linear relation between the paleocortex distance map and inferior-superior gradient level (*r* = 0.67, *P*_spin < 0.001). This suggests that macroscale structural organization varies gradually as a function of paleocortex distance. In contrast, there was negative relationship between inferior and superior proportions of the inferior-superior gradient and archicortex distance (energy test, *P* < 0.02) and a negative, but nonsignificant, linear relationship between this gradient and archicortex distance (*r* = −0.23, *P*_spin > 0.1). We did not observe a consistent association between the dual origin and the posterior-anterior gradient (paleocortex distance: energy test, *P* < 0.001, *r* = −0.22, *P*_spin > 0.1; archicortex: energy test, *P* > 0.1, *r* = −0.02, *P*_spin > 0.1). Evaluating genetic correlation as a function of paleo- and archicortex distance, we observed that genetic correlation varied as a function of distance from both origins.

We performed a similar analysis in macaque monkeys using a previous approximation of the distance from archi- and paleocortex (*34*). The inferior-superior gradient in structural covariance showed a positive association with archicortex distance (energy test, *P* < 0.002, Spearman’s *r* = −0.63, *P*_spin < 0.0001) and a negative association with paleocortex distance (energy test, *P* = ns, Spearman’s *r* = 0.43, *P*_spin < 0.03). Again, we did not observe a consistent association between the dual origin and the posterior-anterior gradient (archicortex distance: energy test, *P* < 0.02, Spearman’s *r* = −0.53, *P*_spin > 0.1; paleocortex distance: energy test, *P* > 0.1, Spearman’s *r* = −0.24, *P*_spin > 0.1).

Last, comparing the covariance gradients in humans against the laminar differentiation as a proxy for sensory-fugal patterning, we observed some correspondence between this model at the main gradients of genetic correlation G1 (Spearman’s *r* = −0.22, *P* < 0.0001) and G2 (Spearman’s *r* = 0.12, *P* < 0.02), although neither survived spin corrections (G1, *P*_spin < 0.1; G2, *P*_spin > 0.1).

## DISCUSSION

Although the importance of macroscale axes of cortical organization for the understanding of human cognition and disease-effects are now recognized, the degree to which these topological features of the cerebral cortex are genetically determined remains incompletely understood. Our current study provided converging evidence that genetic influences contribute to the spatial organization of macroscale structural features of the cortex. In humans, we found two robust topological patterns reflecting organization of thickness; a posterior-anterior and an inferior-superior gradient and almost identical organization patterns were observed when assessing genetic correlation of thickness. We observed similar patterns in macaques, indicating that axes of cortical organization are phylogenetically conserved. Last, we show that, both in humans and macaques, the inferior-superior axis could be aligned with organizational patterns expected based on the dual origin theory, whereas the posterior-to-anterior axis reflected a large-scale functional hierarchy, providing further context to understand the observed topological patterns. Together, these different analyses provide converging evidence of the important role that genetic influences play in macroscale organization of the cortex.

Our study builds on a growing body of evidence describing the organizational axes that determine the patterning of specific brain features such as myeloarchitecture, cytoarchitecture, laminar origin of connections, functional connectivity, cortical thickness, and gene expression (*1*). These studies indicate that the transition from cortical areas with less to more laminar differentiation constitutes sensory-fugal axes across which cortical features systematically vary. These variations have functional and behavioral ramifications (*1*, *28*), and the systematic topological organization of the cerebral cortex has been proposed to optimize the balance of externally and internally oriented functioning, which is critical for flexibility of human cognition (*6*, *7*, *28*). Here, we uncovered two major topological axes in macroscale organization of thickness, of which the posterior-anterior gradient explained the greatest amount of variance in humans. Various studies have demonstrated a posterior-anterior gradient in neuron number in the cortex of a broad range of mammalian species, including rodents, marsupials, and nonhuman primates (*1*, *10*, *36*, *37*). Neuron numbers are high in posterior portions of the cortex, such as the occipital lobe, and gradually decrease toward more anterior regions. The difference in neuronal numbers has been found to relate to the temporal sequence of neurogenesis (*10*, *37*), whereas posterior regions undergo a high number of cell cycles, which accounts for the higher number of neurons in these areas, in anterior regions more time is devoted to the growth of large neurons with many connections (*38*). The posterior-anterior gradient, therefore, might signify a shift in computational capacity, from a high number of processing units in caudal regions to a lower number of highly connected units in rostral regions (*10*). The posterior-anterior organizational gradient had lower than expected values in primary cortices, whereas temporal-parietal regions had higher gradient loadings. This observation aligns with previous work in mammals indicating that both the location along the posterior-anterior axis and an indicator of a region being a primary versus nonprimary area describe variation of neuronal density and synaptic connectivity in mammals best (*39*, *40*). The posterior-anterior covariance gradients showed a positive relation to functional hierarchy, stretching from unimodal to transmodal association cortices (*28*). This puts forward the hypothesis that functional organization arises through genetically driven structural axes and creating an architecture that optimizes the balance of externally and internally oriented functioning, which is critical for flexibility of human cognition. Moreover, it has been suggested that the functional axis described by Margulies *et al*. (*33*) resembles evolutionary adaptation, with regions in the default mode network reflecting a relatively recent evolutionary modification of cortical organization.

Conversely, inferior-superior (dorso-ventral) patterning is extensively described during embryonic development of the central nervous system (*3*, *34*), and dorsal-ventral dichotomies have been reported in macaques (*41*, *42*) and humans (*43*). The inferior-superior axis differentially related to distance from paleo- and archicortex, respectively, aligning the inferior-superior axis in macroscale organization of thickness with the dual origin theory. This convergence supports the view that regions that can be reasonably distant in space can be functionally affiliated because they share similar origins (*3*, *34*, *41*). The emergence of the dual connectional trends might be rooted in two patterning centers in the developing pallium, resulting in two opposing neurogenetic gradients. Both ventral and dorsal systems have been proposed to relate to differentiable functional processes. Whereas the dorsal system has been proposed to relate to time, space, and motility, the ventral system has been associated with assigning meaning and motivation (*43*, *44*). Our analysis only provides correlational evidence of a dual origin schema within cortical macrostructure using distance from archi- and paleocortical formations as a proxy. We hope that the observations reported here will spark further investigation of a dual origin in cortical architecture across imaging modalities and cortical features, as well as its relation to cortical development and ageing.

We observed differential ordering of posterior-anterior and inferior-superior gradients in humans and macaques. Whereas in humans, the principal gradient traversed a posterior-anterior trajectory, we observed that in macaques, this gradient was only the second description of shared variance. This difference might reflect the difference in the timing of cortical development between humans and macaques. For example, it has been shown that in the macaque neurogenesis ends about 20 days earlier in the rostral pole than in the most caudal regions (*45*); in humans, however, a posterior-anterior difference of up to 70 days has been predicted (*37*). It is possible that difference in timing of neurogenesis might describe why the same axis of organization can be more or less pronounced in different species. Previous work, using the same sample of macaques, has shown that similarity in functional cortical organization between humans and macaques decreases with geodesic distance from unimodal systems and culminates in the greater differences in posterior regions of the default network (*33*). It is possible this functional difference emerges from the different balance of the structural organizational patterns between macaques and humans. It has been suggested that the evolution of the shape of the human brain is related to genes involved in neurogenesis and myelination (*46*), resulting in a relatively globular shape of the brain of modern humans relative to their ancestors. It will be important for future work to explore whether differences in the emphasis placed on similar organizational patterns across different species can describe the evolutionary differences in cognitive functions between humans and other primates.

Follow-up analysis indicated the posterior-anterior and inferior-superior gradients related to cortical myelination and previously described organization of microstructural profile covariance (*27*). The posterior-anterior gradient related to T1wT2w contrast in all layers. This is in line with seminal evidence of an increase of mean myelin from fronto-polar toward sensory regions (*47*). The dorsal-ventral dissociation was only observed in the upper two strata, with ventral regions relating to lower T1wT2 contrast than dorsal regions. Difference in upper and lower strata T1wT2w contrast has been summarized using “skewness,” indicating that regions with high difference between upper and lower layers would have a low skewness, whereas regions with a small difference between upper and lower layers having a high skewness (*48*). Dorsal regions including the sensory-motor cortex have been reported to have a low skewness, indicating a large difference in myelin between upper and lower layers. It is possible that the dorsal-ventral patterning of myelin in the upper layers reflects a dissociation in information processing, with sensory agranular regions providing feedforward information and project locally, whereas ventral, more granular paralimbic, regions are involved in feedback processing and project from infragranular layers (*49*). In addition, we found comparable topologies in microstructural profile covariance and macroscale organization of thickness, in line with previous evidence that thickness topology relates to microstructural differentiation (*11*).

Similar to previous work (*13*), we observed a correspondence between organization of structural covariance and geodesic distance. Previous research has indicated that interregional associations, including shared genetic influences, structural covariance, and functional and structural connectivity measures, are more pronounced at short relative to long interregional distances (*13*, *19*). Such a distance effect is consistent with the prediction that evolutionary pressure decreases distances between highly connected brain areas to reduce metabolic and wiring costs (*50*). The spatial constraints on connectivity might relate to signaling molecules, secreted by patterning centers, which generate a graded expression of transcription factors in cortical progenitors (*51*), regulating the position of cortical areas. At the same time, when regressing out distance before assessing the organizational principles underlying structural covariance, we observed patterning that still reflected functional and dual organization, and this time axis followed patterns with in the one hand a juxtaposition of paralimbic areas to heteromodal association cortices, and, on the other hand, a pronounced sensory-fugal trajectory in the second gradient. In addition, other factors shape the organization of brain function and structure such as contralateral homologies and clustering of connections that share inputs (*13*, *52*). Overall, our observations suggest that both distance-related and nondistance-related factors influence the topology of large-scale cortical brain structure, aligning with notions of cortical expansion (*33*), functional hierarchy (*6*), and the dual origin theory (*2*). At the same time, associations between spatial distance and connectivity might be enhanced by motion, smoothing, and measurement error (*53*, *54*). Although our analysis suggests that the observed effects go above and beyond such confounds, further research formally combining multiple modalities and creating mathematical null models randomly capturing topological elements observed in covariance networks (*55*) might help to further decompose the association between geometry and topology of the cortical mantle.

The current work provides a macroscale perspective on the genetic basis of cortical organization by investigating cortical thickness covariance, a widely used, macroscale measure reflecting neuronal density and cytoarchitecture. Cortical structure is defined by not only its thickness but also surface area and gyrification. Future research on the spatial organization of cortical structure might be complemented by models combining multiple cortical features such as cortical thickness, surface area, and folding (*56*, *57*). While implications of our findings in healthy adults to diseased and older populations remain speculative, our work may offer a novel and compelling model to evaluate the impact and progression of pathology. For example, it has been suggested that Parkinson’s and Alzheimer’s disease follows a staging trajectory, with different regions and networks affected at different stages of the disorder (*8*), and its sequence might be determined by underlying anatomical axes. Future work should, therefore, consider whether the macroscale patterns such as those described in our work may shed light on specific orderly sequences in symptoms that underpins Parkinson’s disease, as well as other neurodegenerative conditions. These analyses will not only inform our understanding of the progression of the specific diseases but also provide a model to arrange abnormal features of neurocognitive organization.

To conclude, our results establish two major axes in macroscale organization of cortical thickness in human and nonhuman primates and suggests genetic effects on both. We found a principal gradient stretched from posterior to anterior cortical areas, whereas a second gradient traversed along an inferior-superior axis and aligned with theories on the dual origin of the cortex. Combined, our observations provide direct evidence of a genetic basis behind macroscale patterns of brain structure. Note that our findings were made possible thanks to open data initiatives. These initiatives offer the neuroscience community unprecedented access to large datasets for the investigation of human and nonhuman brains and for the cross-validation of observations across datasets and methods. Uncovering organizational axes of the human cerebral cortex provides insights the developmental-maturational and evolutionary patterns underlying cortical structure. Such axes can be used to study brain-behavior relationships, evaluate disease progression, and disseminate potential neurogenetic origins of abnormal cortical development.

## MATERIALS AND METHODS

### HCP sample

#### Participants and study design

For our analysis, we used the publicly available data from the HCP S1200 release (www.humanconnectome.org/), which comprised data from 1206 individuals (656 females), 298 monozychotic (MZ) twins, 188 dizychotic (DZ) twins, and 720 singletons, with a mean age of 28.8 years (SD, 3.7; range, 22 to 37). We included individuals for whom the scans and data had been released (humanconnectome.org) after passing the HCP quality control and assurance standards. The full set of inclusion and exclusion criteria are described elsewhere (*58*, *59*). In short, the primary participant pool comes from healthy individuals born in Missouri to families that include twins based on data from the Missouri Department of Health and Senior Services Bureau of Vital Records. Additional recruiting efforts were used to ensure that participants broadly reflect ethnic and racial composition of the U.S. population. Healthy is broadly defined to gain a sample generally representative of the population at large. Sibships with individuals having severe neurodevelopmental disorders (e.g., autism), documented neuropsychiatric disorders (e.g., schizophrenia or depression), or neurologic disorders (e.g., Parkinson’s disease) are excluded, as well as individuals with diabetes or high blood pressure. Twins born before 34 weeks of gestation and nontwins born before 37 weeks of gestation are excluded as well. After removing individuals with missing structural imaging data, our sample consisted of 1113 (606 females) individuals (including 286 MZ twins and 170 DZ twins) with a mean age of 28.8 years (SD, 3.7; range, 22 to 37).

#### Structural imaging processing

Magnetic resonance imaging (MRI) protocols of the HCP are previously described (*58*, *59*). In short, MRI data used in the study were acquired on the HCP’s custom 3T Siemens Skyra equipped with a 32-channel head coil. Two T1w images with identical parameters were acquired using a three-dimensional magnetization-prepared rapid gradient-echo (3D MP-RAGE) sequence (0.7-mm isotropic voxels; matrix, 320 × 320; 256 sagittal slices; common MRI setting terms. Two T2w images were acquired using a 3D T2-SPACE sequence with identical geometry (TR, 3200 ms; TE, 565 ms; variable flip angle; iPAT, 2). T1w and T2w scans were acquired on the same day. The pipeline used to obtain the FreeSurfer segmentation is described in detail in a previous article (*58*) and is recommended for the HCP data. The preprocessing steps included coregistration of T1- and T2-weighted scans, B1 (bias field) correction, and segmentation and surface reconstruction using FreeSurfer version 5.3 HCP to estimate cortical thickness.

In addition to assess robustness and replicability of the results across different surface estimation pipelines, cortical thickness estimates were further estimated using FreeSurfer version 6.0 and CIVET (*60*). For both these additional analyses, only bias-corrected T1-weighted data were used as the input. FreeSurfer version 6.0 was performed using the default recon-all options. Surface-extraction and cortical thickness estimation using CIVET were performed using version 2.1.1 (www.bic.mni.mcgill.ca/ServicesSoftware/CIVET). The nonuniformity artefacts were corrected with the N3 algorithm using the recommended N3 spline distance of 125 mm for 3T T1-weighted scans. Cortical thickness was then measured as the distance between the estimated “white” and “gray” cortical surfaces, in the native space framework of the original MR images, using the same approach that is used in FreeSurfer.

#### Parcellation approach

We used the Schaefer parcellation scheme (*25*) on the basis of the combination of a local gradient approach and a global similarity approach using a gradient-weighted Markov Random models. The parcellation has been extensively evaluated with regard to stability and convergence with histological mapping and alternative parcellations. In the context of the current study, we focus on the granularity of 400 parcels, as averaging will improve signal to noise. We averaged unsmoothed structural data within each parcel. Thus, cortical thickness of each region of interest was estimated as the trimmed mean (10% trim). Findings were additionally evaluated using different parcellation schemes using the 800 parcel Schaefer (*25*) solution, as well as the Glasser atlas (*31*) and the Desikan-Killiany (*30*) atlas.

#### Structural covariance

We computed structural covariance by correlating cortical thickness parcels while controlling for age, sex, and global thickness, resulting in a 400 by 400 matrix. Previous work has indicated that there is a strong general genetic component influencing cortical anatomy (*61*), and thus, by regressing out global thickness, these global genetic effects are reduced. However, the main observations remain virtually identical when not controlling for global thickness.

#### Genetic correlation analysis

To investigate the genetic correlation of brain structure, we analyzed 400 parcels of cortical thickness in a twin-based genetic correlation analysis. The quantitative genetic analyses were conducted using SOLAR (*62*). SOLAR uses maximum likelihood variance-decomposition methods to determine the relative importance of familial and environmental influences on a phenotype by modeling the covariance among family members as a function of genetic proximity. We used a G + E model to assess heritability and genetic correlation in the HCP dataset on the basis of prior work, indicating that G + E is more parsimonious and leads to more reproducible results in this sample (*63*). This approach can handle pedigrees of arbitrary size and complexity and thus is optimally efficient with regard to extracting maximal genetic information. To ensure that our cortical thickness parcels were conforming to the assumptions of normality, an inverse normal transformation was applied.

Heritability (*h*^2^) represents the portion of the phenotypic variance (σ^2^_p_) accounted for by the total additive genetic variance (σ^2^_g_), i.e., *h*^2^ = σ^2^_g_/σ^2^_p_. Phenotypes exhibiting stronger covariances between genetically more similar individuals than between genetically less similar individuals have higher heritability. Within SOLAR, this is assessed by contrasting the observed covariance matrices for a neuroimaging measure with the structure of the covariance matrix predicted by kinship. Heritability analyses were conducted with simultaneous estimation for the effects of covariates. For this study, we included covariates including global thickness, age, sex, age^2^, and age × sex.

To determine whether shared variations in cortical thickness were influenced by the same genetic factors, genetic correlation analyses were conducted. More formally, bivariate polygenic analyses were performed to estimate genetic (ρ_g_) and environmental (ρ_e_) correlations, on the basis of the phenotypic correlation (ρ_p_), between cortical thickness parcels in the following formula: $\rho_{p}=\rho_{g}\sqrt{\left({h^{2}}_{1}{h^{2}}_{2}\right)}+\rho_{e}\sqrt{\left[(1-{h^{2}}_{1})(1-{h^{2}}_{2})\right]}$, where *h*^2^_1_ and *h*^2^_2_ are the heritability of the parcel-based cortical thickness. The significance of these correlations was tested by comparing the log likelihood for two restricted models (with either ρ_g_ or ρ_e_ constrained to be equal to 0) against the log likelihood for the model in which these parameters were estimated. A significant genetic correlation is evidence suggesting that (a proportion of) both phenotypes are influenced by a gene or set of genes (*64*). To compute the contribution of genetic effects relative to the phenotypic correlation, we computed the contribution of the genetic path to the phenotypic correlation $\left(\sqrt{{h^{2}}_{1}}\times \rho_{g}\times \sqrt{{h^{2}}_{2}}\right)$ (ρ_ph_g) divided by the phenotypic correlation. For the relative contribution of environmental correlation to the phenotypic correlation, we computed $\left(\sqrt{1-{h^{2}}_{1}}\times \rho_{e}\times \sqrt{1-{h^{2}}_{2}}\right)$ (ρ_ph_e) divided by the phenotypic correlation (*65*).

#### Gradient decomposition

To compute macroscale organizational gradients, we performed several analysis steps. The input of the analysis was the structural covariance/genetic correlation matrix, which was cut off at 90% similar to previous studies (*27*, *28*). To study the relationships between cortical regions in terms of their features, we used a normalized angle similarity kernel resulting in a non-negative square symmetric affinity matrix. In the following, we used diffusion mapping, a nonlinear dimensionality reduction method (*29*). In brief, the algorithm estimates a low-dimensional embedding from a high-dimensional affinity matrix. In this space, cortical nodes that are strongly interconnected by either many suprathreshold edges or few very strong edges are closer together, whereas nodes with little or no covariance are farther apart. The name of this approach, which belongs to the family of graph Laplacians, derives from the equivalence of the Euclidean distance between points in the embedded space and the diffusion distance between probability distributions centered at those points. It is controlled by a single parameter α, which controls the influence of the density of sampling points on the manifold (α = 0, maximal influence; α = 1, no influence). On the basis of the previous work (*27*, *28*), we followed recommendations and set α = 0.5, a choice that retains the global relations between data points in the embedded space and has been suggested to be relatively robust to noise in the covariance matrix. Gradients were mapped onto the cortical surface using SurfStat (http://mica-mni.github.io/surfstat), and we assessed the amount of variance explained.

#### Functional connectivity

Functional connectivity matrices were based on 1 hour of resting-state functional MRI (fMRI) data acquired through the HCP (*58*) and made publicly available for download on ConnectomeDB. Functional resting-state MRI data underwent HCP’s minimal preprocessing (*31*, *58*). Briefly, for each individual, a functional connectivity matrix was calculated using the correlation coefficient across four minimally preprocessed, spatially normalized, and concatenated to four 15-min resting-state fMRI scans and coregistered using MSMAll to template HCP 32k_LR surface space (*59*). 32k_LR surface space consists of 32,492 total nodes per hemisphere (59,412 excluding the medial wall). Following average time series were extracted in each of the 400 cortical parcels (*25*), and individual functional connectivity matrices were computed. The individual functional connectomes were generated by averaging preprocessed time series within nodes, correlating nodal time series, and converting them to *z* scores. Using the individual time series of individuals with complete data in the S1200 sample, we constructed an average functional connectivity matrix of which we derived a principle gradient.

#### Geodesic distance

Geodesic distance was measured as the length of the shortest path between two points (i.e., two surface vertices) running through the cortical mantle using an approach invariant to mesh configuration (*66*). Geodesic distance was computed between each vertex in fsaverage5 space using the Eucledian vertex coordinates, creating a 20,484 × 20,484 distance matrix. Only ipsilateral distance was considered. Next, distances between parcels were computed by taking the average distance between both parcels. To assess the association between structural covariance and distance in humans and macaques, we computed gradients based on the geodesic distance within each cortical hemisphere. Using these gradients, we probed genetic correlation of thickness along the distance-based gradients. To assess the spatial organization of covariance while controlling for distance, we used a linear regression approach and regressed the geodesic distance and geodesic distance^2^ between parcels from their respective covariance. Next, we computed the principal gradients of the distance-regressed covariance and evaluated its relationship to the various models of cortical organization.

#### Comparisons between gradients and modalities

To make comparisons across gradient and distance maps, we used spin tests to control for spatial autocorrelation when possible (*55*). Difference between two distributions was assessed using statistical energy test, a nonparametric statistic for two sample comparisons (*35*) (https://github.com/brian-lau/multdist/blob/master/minentest.m), and statistical significance was assessed with permutation tests (1000). Thus, spin tests are used to assess significances of similarity of continuous spatial maps that have spatial autocorrelations, whereas statistical energy tests were used to compare two distributions.

#### Macaque sample

We used the MRI data from the recently formed non-human primate (NHP) data sharing consortium PRIME-DE (http://fcon_1000.projects.nitrc.org/indi/indiPRIME.html). Three cohorts of macaque monkeys were included in the present study (Newcastle University, Oxford University, and University of California, Davis).

### Oxford data

The full dataset consisted of 20 rhesus macaque monkeys (*Macaca mulatta*) scanned on a 3T scanner with four-channel coil. The data were collected while the animals were under anesthesia. Briefly, the macaque was sedated with intramuscular injection of ketamine (10 mg/kg) combined with either xylazine (0.125 to 0.25 mg/kg) or midazolam (0.1 mg/kg) and buprenorphine (0.01 mg/kg). In addition, macaques received injections of atropine (0.05 mg/kg, intramuscularly), meloxicam [0.2 mg/kg, intravenously (i.v.)], and ranitidine (0.05 mg/kg, i.v.). The anesthesia was maintained with isoflurane. The details of scan and anesthesia procedures are described in (*67*) and the PRIME-DE website (http://fcon_1000.projects.nitrc.org/indi/PRIME/oxford.html). Protocols for animal care, MRI, and anesthesia were carried out under authority of personal and project licenses in accordance with the UK Animals (Scientific Procedures) Act (1986) (*67*, *68*).

### UC Davis data

The full dataset consisted of 19 rhesus macaque monkeys (*M. mulatta*, all female; age, 20.38 ± 0.93 years; weight, 9.70 ± 1.58 kg) scanned on a Siemens Skyra 3T with four-channel clamshell coil. All the animals were scanned under anesthesia. In brief, the macaques were sedated with injection of ketamine (10 mg/kg), dexmedetomidine (0.01 mg/kg), and buprenorphine (0.01 mg/kg). The anesthesia was maintained with isoflurane at 1 to 2%. The details of the scan and anesthesia protocol can be found at http://fcon_1000.projects.nitrc.org/indi/PRIME/ucdavis.html. The neuroimaging experiments and associated procedures were performed at the California National Primate Research Center under protocols approved by the University of California, Davis Institutional Animal Care and Use Committee (*69*).

### Newcastle data

The full dataset consisted of 14 rhesus macaque monkeys (*M. mulatta*) scanned on a Vertical Bruker 4.7T primate dedicated scanner. We restricted our analysis to 10 animals (8 males; age, 8.28 ± 2.33; weight, 11.76 ± 3.38) for whom two awake resting-state fMRI scans were required. The structural T1-weighted images were acquired using MDEFT sequence with resolution of 0.6 mm by 0.6 mm by 0.6 mm; TE, 6 ms; and TR, 750 ms. All nonhuman animal scans and associated procedures were performed at Newcastle University, UK and were approved by the Animal Welfare and Ethical Review Body at Newcastle University and by the UK Home Office (*70*).

### MRI data processing

The structural processing includes (i) spatial denoising by a nonlocal mean filtering operation (*71*), (ii) brain extraction using advanced normalization tools registration with a reference brain mask followed by manually editing to fix the incorrect volume (ITK-SNAP, www.itksnap.org) (*72*), (iii) tissue segmentation and surface reconstruction (FreeSurfer) (*73*), and (iv) the native white matter and pial surfaces were registered to the Yerkes19 macaque surface template (*74*).

#### Quality control

We excluded macaque monkeys that showed a hemispheric difference of more than 0.2 cm [UC Davis (0) Oxford (7), and Newcastle (5)] for our final analysis, as gradient models were estimated on the basis of covariance of ipsi- and contralateral covariance.

#### Gradient analysis

First, we constructed a covariance matrix, controlling for dataset site and global thickness. Next, we performed gradient analysis analog to described in humans.

#### Alignment of human gradients to macaque gradients

To evaluate the similarity between human and macaque gradients, we transformed the human gradient to macaque cortex based on a functional alignment techniques recently developed. This method leverages advances in representing functional organization in high-dimensional common space and provides a transformation between human and macaque cortices (*33*).

#### Archicortex and paleocortex distance

Distance from the archicortex and paleocortex was computed in Goulas *et al*. (*34*).

#### Replication sample: eNKI

##### Participants and study design

To evaluate the cross-sample reproducibility of observations, we additionally investigated cortical thickness covariance in the enhanced Nathan Kline Institute-Rockland Sample (NKI). The sample was made available by the Nathan-Kline Institute (NKY, NY, USA) (*75*). In short, eNKI was designed to yield a community-ascertained, life span sample in which age, ethnicity, and socioeconomic status are representative of Rockland County, NY, USA. ZIP code–based recruitment and enrollments efforts were being used to avoid overrepresentation of any portion of the community. Participants below 6 years were excluded to balance data losses with scientific yield, as well as participants above the age of 85, as chronic illness was observed to markedly increase after this age. All approvals regarding human subjects’ studies were sought following NKI procedures. Scans were acquired from the International Neuroimaging Data Sharing Initiative (INDI) online database (http://fcon_1000.projects.nitrc.org/indi/enhanced/studies.html). For our phenotypic analyses, we selected individuals with complete cortical thickness data. Our sample for phenotypic correlations consisted of 799 (400 females) individuals with a mean age of 41.1 years (SD, 20.3; range, 12 to 85).

##### Structural imaging processing

The 3D MP-RAGE imaging structural scans were acquired using a 3.0T Siemens Trio scanner with TR of 2500 ms, TE of 3.5 ms; bandwidth of 190 Hz/Px, field of view of 256 mm by 256 mm, flip angle of 8°, and voxel size of 1.0 mm by 1.0 mm by 1.0 mm. More details on image acquisition are available at http://fcon_1000.projects.nitrc.org/indi/enhanced/studies.html. All T1 scans were preprocessed using the FreeSurfer software library (https://surfer.nmr.mgh.harvard.edu) version 6.0.0 (*74*) to compute cortical thickness. Next, the individual cortical thickness and surface area maps were standardized to fsaverage5 for further analysis. Segmentations were visually inspected for anatomical errors (S.L.V.).

#### Cortical thickness methodology

Cortical thickness estimates of the individuals of the HCP S1200 release using differing processing pipelines were computed as part of an independent study (*60*) and resampled to Schaefer 400 parcels. We used the extracted thickness values of FreeSurfer 6.0 to evaluate the stability of observed covariance organization as a function of cortical thickness estimation method. For the FreeSurfer 6.0 analysis of the T1-weighted images in the HCP dataset, we used the default recon-all options (version 6.0; https://surfer.nmr.mgh.harvard.edu). Moreover, cortical thickness estimation using CIVET was performed using version 2.1.1 (www.bic.mni.mcgill.ca/ServicesSoftware/CIVET).

#### Cortical microstructure and microstructural covariance networks

We estimated microstructural profile covariance (MPC) using myelin-sensitive MRI, in line with the previously reported protocol (*27*), in the S900 HCP sample. The myelin-sensitive contrast was T1w/T2w from the HCP minimal processing pipeline, which uses the T2w to correct for inhomogeneities in the T1w image. We generated 12 equivolumetric surfaces between the outer and inner cortical surfaces. The equivolumetric model compensates for cortical folding by varying the Euclidean distance ρ between pairs of intracortical surfaces throughout the cortex to preserve the fractional volume between surfaces. ρ was calculated as follows for each surface Eq. 1

$$\rho =\frac{1}{A_{\text{out}}-A_{\text{in}}}.(-A_{\text{in}}+\sqrt{\alpha A_{\text{out}}^{2}+(1-\alpha )A_{\text{in}}^{2}})$$ (1)

where α represents a fraction of the total volume of the segment accounted for by the surface, while *A*_out_ and *A*_in_ represents the surface area of the outer and inner cortical surfaces, respectively. We systematically sampled T1w/T2w values along 64,984 linked vertices from the outer to the inner surface across the whole cortex. Subsequently, we computed the average value of T1w/T2 in each of the 400 parcels of the Schaefer atlas (*25*). In turn, MPC_MRI_(*i*, *j*) for a given pair of parcels *i* and *j* is defined in Eq. 2

$${\text{MPC}}_{\text{MRI}}(i,j)=\frac{1}{n}\Sigma_{s=1}^{n}{\left(\frac{r_{\mathrm{ij}}-r_{\mathrm{ic}}r_{\mathrm{jc}}}{\sqrt{\left(1-r_{\mathrm{ic}}^{2})(1-r_{\mathrm{jc}}^{2}\right)}}\right)}_{s}$$ (2)

where *s* is a participant and *n* is the number of participants. We used the MPC_MRI_ to compute the gradient of microstructure.

### Data availability

This study followed the institutional review board guidelines of corresponding institutions. All human data analyzed in this manuscript were obtained from the open-access HCP young adult sample (HCP; www.humanconnectome.org/) (*59*) and eNKI (www.ncbi.nlm.nih.gov/pmc/articles/PMC3472598/) (*75*). Scans were acquired from the INDI online database (http://fcon_1000.projects.nitrc.org/indi/enhanced/studies.html). The raw data may not be shared by third parties due to ethics requirements but can be downloaded directly via the above web links. Macaque data were obtained from the recently formed NHP data sharing consortium PRIME-DE (http://fcon_1000.projects.nitrc.org/indi/indiPRIME.html). Three cohorts of macaque monkeys were included in the present study (Newcastle University, Oxford University, and University of California, Davis). Genetic analyses were performed using Solar Eclipse 8.4.0 (www.solar-eclipse-genetics.org), and data on the KING pedigree analysis are available at https://www.nitrc.org/projects/se_linux/ (*62*, *76*). Gradient mapping analyses were based on open-access tools (BrainMap, https://brainspace.readthedocs.io/en/latest/). Surface-wide statistical comparisons and visualizations were carried out using SurfStat (https://github.com/MICA-MNI/micaopen/tree/master/surfstat) in combination with ColorBrewer (https://github.com/scottclowe/cbrewer2). Both structural covariance and genetic correlation gradients are available at https://github.com/sofievalk/projects/tree/master/Structure_of_Structure.

## Supplementary Material

abb3417_SM.pdf

## SUPPLEMENTARY MATERIALS

Supplementary material for this article is available at http://advances.sciencemag.org/cgi/content/full/6/39/eabb3417/DC1

## Appendix

View/request a protocol for this paper from *Bio-protocol*.
