# Supplementary material for: Shaping brain structure: Genetic and phylogenetic axes of macroscale organization of cortical thickness
Source: Sci Adv. 2020 Sep 25;6(39):eabb3417. doi: 10.1126/sciadv.abb3417 (PMC7518868; doi:10.1126/sciadv.abb3417)
Supplement: abb3417_SM.pdf [file abb3417_SM.pdf]

Science Advances
AAAS

advances.sciencemag.org/cgi/content/full/6/39/eabb3417/DC1

Supplementary Materials for

Shaping brain structure: Genetic and phylogenetic axes of macroscale organization of cortical thickness

Sofie L. Valk*, Ting Xu, Daniel S. Margulies, Shahrzad Kharabian Masouleh, Casey Paquola, Alexandros Goulas, Peter Kochunov, Jonathan Smallwood, B. T. Thomas Yeo, Boris C. Bernhardt, Simon B. Eickhoff

*Corresponding author. Email: s.valk@fz-juelich.de

Published 25 September 2020, *Sci. Adv.* **6**, eabb3417 (2020)
DOI: 10.1126/sciadv.abb3417

This PDF file includes:

Supplementary Results
Tables S1 and S2
Figs. S1 to S8
References

Supplementary Results

Replication of structural covariance gradients in eNKI dataset

To evaluate whether the observed organizational axes of structural covariance could also be detected in a different dataset with a wider age-range, we evaluated the structural covariance gradients in the eNKI dataset (799 individuals, ages 12-85yrs). Here we observed, similar to the main observations in the HCP dataset, a principal anterior posterior gradient explaining 15% of variance and a secondary gradient traversing from inferior to superior regions explaining 11% of variance. Overall patterns were highly comparable (G1: $r=0.81$, $p_{spin}<0.001$, G2: $r=0.88$, $p_{spin}<0.001$) between HCP and eNKI covariance gradients (**Fig S1**).

Robustness of principal and secondary gradient

To assess robustness we compared the first two gradients from our main analysis relative to the first two gradients when varying various analysis steps in the gradient computation, exploring different algorithm parameters through the BrainSpace toolbox (*29*). Other kernels (Pearson, Spearman, Gaussian, or Cosine Similarity) showed all a high correlation between the original G1 and G2 and their respective outputs ($r>0.90$). Also, the use of an alternative non-linear dimension reduction (Laplacian eigenmap) or linear approach (PCA) give highly similar results ($r>0.95$). Last, varying the cut-off value of the covariance matrix did not change the outcome ($r>0.90$).

The third – eight gradient of thickness covariance and genetic correlation of thickness.

Additionally, we studied the third-eight gradient of thickness covariance and genetic correlation of thickness, explaining 5-10% of variance (**Fig S3**). The third gradient traversed from sensory-motor and mid temporal areas to both frontal and occipital cortices, and a comparable gradient was observed in genetic correlation of thickness. The fourth gradient had a bilateral axis in superior dorsolateral frontal cortex on the one hand and frontal polar, parietal and temporal

polar regions on the other hand. The fifth gradient showed strong lateralization between left temporal parietal regions and right lingual gyrus and corresponded to the sixth gradient of genetic correlation of thickness. The sixth gradient was centered in the right supramarginal gyrus extending to sensory-motor areas on the one hand, and less so in the left sensory cortex, and on the other hand precuneus and para-limbic areas, a similar gradient was not observed in genetic correlation of thickness. The seventh gradient related to sensory-motor, fusiform gyrus and posterior-mid cingulate on the one hand, and temporal regions and precuneus on the other and was most pronounced in the right hemisphere, this gradient was similar to the fifth gradient in coheritability of thickness. The eighth gradient showed a dissociation between temporal parietal regions and posterior-mid cingulate on the one hand, and occipital and sensory regions on the other.

Structural gradients are above and beyond geodesic distance.

Previous work has shown a strong relationship between structural thickness covariance, genetic correlation of cortical thickness, and geodesic distance. Thus, we explored the relationship between organization of structural covariance and geodesic distance. Geodesic distance was defined as the average distance between each of the 400 parcels ipsilaterally (**Fig S6**). In line with previous reports, we observed a strong relation between structural covariance and geodesic distance (left hemisphere: $r=-0.52$, $p<0.00001$, right hemisphere: $r=-0.51$, $p<0.00001$). Moreover, we observed that genetic correlation varied as a function of the organization of distance, with regions at comparable levels of the geodesic distance gradients showing high genetic correlation among each other. The current work capitalized on unsupervised assessments of large-scale cortical organizational patterns capturing main global embedding axes based on each regions' covariance profile. Given that visual and prefrontal cortex are most distant from each other, this is the dominant large-scale axis based on geodesic distance.

When regressing out the effects of geodesic distance on structural covariance we found evidence suggesting that the principal gradients of structural covariance remain largely intact, yet reversed (G1: $G1_{DISTCOV}$: r= 0.13, p>0.05; $G2_{DISTCOV}$: r=0.65, p_spin<0.01 and G2: $G_{DISTCOV}$: r=0.80, p_spin<0.001; $G2_{DISTCOV}$: r=-0.34, p_spin>0.1). The primary covariance gradient stretched from sensory-motor, temporal, and paralimbic regions to frontal and parietal cortices, whereas the secondary gradient stretched from visual and sensory-motor cortex to default and paralimbic areas. Post-hoc analysis suggested that the first (archicortex: r=-0.13, p=0.07 (p_spin >0.1), energy-test: p<0.1, and paleocortex: r=0.50, p<0.0001 (p_spin<0.002), energy-test: p<0.001) but not the second (archicortex: r=0.01, energy p>0.1 and paleocortex: r=-0.57, p<0.0001 (p_spin<0.002), energy-test: p<0.001), gradient showed a differential relation to archi- and paleocortex distance, albeit at trend level. Moreover, the second (r=0.53, p_spin<0.005) but not the first (r=0.24, p_spin>0.1) gradient related to functional organization. Comparing the distance-corrected covariance gradients to laminar differentiation, we found that while the second gradient traversed from paralimbic to idiotypic areas (Spearman's r=-0.47, p<0.0001 (p_spin<0.005)), while the principal gradient did not (Spearman's r=-0.03, p>0.1), but rather highlighted a differential mapping of paralimbic versus heteromodal areas.

Regressing out effects of distance in covariance in macaques, we observed a correspondence between the principal gradients of structural covariance and the distance-corrected gradients (G1: $G1_{DISTCOV}$: r= 0.83, p_spin<0.001; $G2_{DISTCOV}$: r=0.07, p>0.1 and G2: $G_{DISTCOV}$: r=-0.08, p>0.1; $G2_{DISTCOV}$:r=0.68, p_spin<0.01), as well as a trend-level association of the first (paleocortex: Spearman's r=0.44, p<0.02, energy-test p<0.1, archicortex: r=-0.27, p>0.1, energy-test p<0.1) but not the second (paleocortex: Spearman's r=-0.07, archicortex: Spearman's r=-0.03) gradient with the dual origin model. Last, comparing the human and macaque distance-corrected covariance gradients, we found a correspondence between the principal gradient in both species (r=0.38, p<0.0005) and the second gradient in both species

(r=0.29, p<0.005), but not across gradients ($G1_{macaque}$-$G2_{human}$: r=-0.07; $G2_{macaque}$-$G1_{human}$: r=0.11).

Relationship between large-scale organization of genetic correlation of regional thickness and microstructure profiles

In a last step we evaluated the association between the two main axis of regional covariance topology and cortical microstructure (T1w/T2w) and microstructural covariance gradients (*27*), in order to qualify and quantify the relation of the observed covariance gradients in thickness to previously reported microstructural organization (*27*). We probed cortical microstructure at 12 equidistant surfaces sampled between the outer and inner cortical layer in a sub-set of our participants (HCP S900 sample). We observed a strong negative relationship between $G1_{scov}$ and cortical T1w/T2w at all layer depts (-0.34 < r >-0.44) (**Fig S7; Table S2**). $G2_{scov,}$ however, only showed a significant positive association with the two most outer strata (layer 1: r=0.60, layer 2: r=0.40), but not with layers closer to the GM/WM surface (**Fig S7; Table S2**). Subsequently, we probed the association between organizational gradients of within-individual microstructural profile covariance and topological organization of structural covariance of cortical thickness. To do so, we computed the mean microstructural profile covariance (MPC) maps across individuals and preformed gradient decomposition. We observed, as previously reported (*27*) a primary gradient of cortical microstructural profile covariance traversing a sensory-fugal pattern (22% of variance), and secondary gradient (17% of variance) traversing a pattern from sensory-motor to frontal cortices. We found that the first MPC gradient showed a close correlation with the inferior-superior gradient of genetic covariance of thickness (r=0.62, p_spin<0.005), but not with the posterior-anterior gradient of genetic covariance of thickness (r=-0.02). Conversely, the secondary gradient of MPC was associated with the posterior-anterior gradient of genetic covariance of thickness (r=0.30, p_spin<0.025), but not with the inferior-superior gradient of genetic covariance (r=-0.09, p>0.1).

Functional topography along macro-scale organizational patterns of thickness

We conducted a meta-analysis using the Neurosynth (*78*) database and estimated the center of gravity across a set of diverse cognitive terms (*27, 28*) along the posterior-anterior and inferior-superior macro-scale organization patterns of thickness (**Fig S8**). In the posterior-anterior gradient we observed a divergence between sensory and visual functions posteriorly and 'working-memory', 'reading', as well as 'motor' and 'action' processing anteriorly. Various terms such as 'emotion' and 'reward' related to both posterior and anterior regions. The inferior-superior gradient on the other hand related to 'motor', 'working memory' and 'action' in superior regions, but 'emotion', 'reward', 'affective', 'pain' in inferior regions.

SUPPLEMENTARY TABLE

ROI	h2	p	ROI	h2	p	ROI	h2	p	ROI	h2	p
**1**	0,223	5,50E-06	**101**	0,398	1,76E-06	**201**	0,246	5,00E-07	**301**	0,143	5,86E-03
**2**	0,379	2,93E-13	**102**	0,379	3,97E-05	**202**	0,440	1,48E-08	**302**	0,395	4,79E-06
**3**	0,205	6,57E-05	**103**	0,247	4,20E-06	**203**	0,183	5,75E-04	**303**	0,249	3,50E-06
**4**	0,358	7,01E-14	**104**	0,331	1,68E-02	**204**	0,217	2,17E-05	**304**	0,315	3,27E-01
**5**	0,358	3,54E-12	**105**	0,118	2,33E-02	**205**	0,378	8,46E-07	**305**	0,100	3,90E-02
**6**	0,223	1,69E-05	**106**	0,334	3,45E-04	**206**	0,370	6,15E-05	**306**	0,347	5,77E-04
**7**	0,289	6,13E-10	**107**	0,347	8,85E-04	**207**	0,174	6,36E-04	**307**	0,338	4,10E-02
**8**	0,116	1,67E-02	**108**	0,296	1,00E-07	**208**	0,381	6,68E-06	**308**	0,352	3,18E-03
**9**	0,387	3,42E-13	**109**	0,233	2,96E-05	**209**	0,229	1,65E-05	**309**	0,189	2,46E-04
**10**	0,436	3,46E-11	**110**	0,284	2,00E-07	**210**	0,176	5,36E-04	**310**	0,318	1,05E-02
**11**	0,356	1,78E-04	**111**	0,228	1,42E-05	**211**	0,425	1,07E-07	**311**	0,294	1,00E-07
**12**	0,452	1,04E-09	**112**	0,364	6,40E-05	**212**	0,519	1,12E-17	**312**	0,196	1,90E-04
**13**	0,331	4,31E-03	**113**	0,178	4,24E-04	**213**	0,414	1,25E-05	**313**	0,300	3,55E-01
**14**	0,163	1,06E-03	**114**	0,431	9,18E-09	**214**	0,471	4,15E-12	**314**	0,240	1,44E-05
**15**	0,411	4,28E-07	**115**	0,291	1,00E-07	**215**	0,387	1,87E-05	**315**	0,360	4,64E-05
**16**	0,496	1,05E-13	**116**	0,373	5,34E-05	**216**	0,122	1,01E-02	**316**	0,328	2,14E-02
**17**	0,172	7,86E-04	**117**	0,302	1,00E-07	**217**	0,520	8,72E-11	**317**	0,217	3,41E-05
**18**	0,402	5,40E-09	**118**	0,452	2,23E-09	**218**	0,348	5,06E-04	**318**	0,231	4,28E-05
**19**	0,448	1,66E-12	**119**	0,176	7,01E-04	**219**	0,594	1,35E-21	**319**	0,342	4,54E-04
**20**	0,468	8,71E-13	**120**	0,185	2,37E-04	**220**	0,500	2,42E-12	**320**	0,231	5,10E-06
**21**	0,528	6,94E-17	**121**	0,350	3,08E-02	**221**	0,382	8,37E-05	**321**	0,464	1,18E-11
**22**	0,406	7,06E-07	**122**	0,278	1,00E-07	**222**	0,204	4,30E-05	**322**	0,278	3,00E-07
**23**	0,338	1,18E-02	**123**	0,337	1,01E-03	**223**	0,452	1,91E-09	**323**	0,245	1,06E-05
**24**	0,385	8,46E-05	**124**	0,227	2,93E-05	**224**	0,445	1,41E-10	**324**	0,298	1,34E-01
**25**	0,457	7,53E-11	**125**	0,306	4,97E-03	**225**	0,390	5,06E-08	**325**	0,196	2,34E-04
**26**	0,485	4,67E-12	**126**	0,525	5,96E-15	**226**	0,333	2,11E-02	**326**	0,303	2,00E-07
**27**	0,302	9,82E-03	**127**	0,243	3,60E-06	**227**	0,217	1,03E-04	**327**	0,206	1,27E-04
**28**	0,191	2,65E-04	**128**	0,176	3,48E-04	**228**	0,324	7,59E-04	**328**	0,349	2,84E-04
**29**	0,511	1,73E-13	**129**	0,161	2,09E-03	**229**	0,411	2,25E-07	**329**	0,305	6,85E-02
**30**	0,350	5,85E-06	**130**	0,257	4,40E-06	**230**	0,302	1,57E-01	**330**	0,243	5,80E-06
**31**	0,422	1,85E-07	**131**	0,134	6,76E-03	**231**	0,490	3,12E-11	**331**	0,480	5,52E-13
**32**	0,468	4,83E-10	**132**	0,090	4,75E-02	**232**	0,245	3,40E-06	**332**	0,144	2,78E-03
**33**	0,286	2,61E-01	**133**	0,048	1,93E-01	**233**	0,327	1,55E-02	**333**	0,217	3,13E-05
**34**	0,275	1,00E-07	**134**	0,274	1,00E-07	**234**	0,422	2,66E-07	**334**	0,096	4,35E-02
**35**	0,226	4,05E-05	**135**	0,277	3,00E-07	**235**	0,200	4,28E-04	**335**	0,142	6,71E-03
**36**	0,430	4,30E-08	**136**	0,207	6,02E-05	**236**	0,347	2,97E-03	**336**	0,171	6,06E-04
**37**	0,109	3,04E-02	**137**	0,190	2,48E-04	**237**	0,219	3,63E-05	**337**	0,080	6,30E-02
**38**	0,195	1,27E-04	**138**	0,342	1,40E-03	**238**	0,129	1,03E-02	**338**	0,161	1,46E-03
**39**	0,349	8,35E-03	**139**	0,235	3,20E-06	**239**	0,330	1,24E-02	**339**	0,123	1,59E-02
**40**	0,317	2,54E-02	**140**	0,350	9,33E-06	**240**	0,322	3,81E-02	**340**	0,251	2,60E-06

ROI	h2	p	ROI	h2	p	ROI	h2	p	ROI	h2	p
**41**	0,210	4,81E-05	**141**	0,191	2,58E-04	**241**	0,309	3,96E-01	**341**	0,190	4,62E-04
**42**	0,215	3,70E-05	**142**	0,206	7,64E-05	**242**	0,180	5,18E-04	**342**	0,163	1,04E-03
**43**	0,276	2,00E-07	**143**	0,250	4,10E-06	**243**	0,102	3,33E-02	**343**	0,354	1,50E-04
**44**	0,424	5,94E-09	**144**	0,372	2,34E-05	**244**	0,243	9,30E-06	**344**	0,275	1,00E-07
**45**	0,294	1,06E-01	**145**	0,323	4,51E-03	**245**	0,164	7,11E-04	**345**	0,245	5,00E-06
**46**	0,360	4,09E-05	**146**	0,396	5,79E-05	**246**	0,477	1,74E-12	**346**	0,306	1,60E-01
**47**	0,221	1,82E-05	**147**	0,509	3,42E-13	**247**	0,285	2,95E-01	**347**	0,246	4,70E-06
**48**	0,367	1,45E-05	**148**	0,315	4,05E-02	**248**	0,271	1,00E-07	**348**	0,224	2,79E-05
**49**	0,253	3,70E-06	**149**	0,192	2,38E-04	**249**	0,396	3,40E-07	**349**	0,312	1,15E-01
**50**	0,296	2,44E-01	**150**	0,248	1,60E-06	**250**	0,232	2,16E-05	**350**	0,211	3,94E-05
**51**	0,462	1,83E-11	**151**	0,225	5,90E-06	**251**	0,529	4,57E-17	**351**	0,314	4,65E-03
**52**	0,299	1,03E-01	**152**	0,179	4,16E-04	**252**	0,258	1,30E-06	**352**	0,230	1,57E-05
**53**	0,143	3,31E-03	**153**	0,335	5,15E-02	**253**	0,268	9,00E-07	**353**	0,056	1,39E-01
**54**	0,397	3,46E-06	**154**	0,256	8,00E-07	**254**	0,305	4,56E-02	**354**	0,320	2,90E-03
**55**	0,222	3,35E-05	**155**	0,098	3,06E-02	**255**	0,305	1,70E-01	**355**	0,197	1,88E-04
**56**	0,156	1,80E-03	**156**	0,281	1,00E-01	**256**	0,436	2,67E-08	**356**	0,264	4,00E-07
**57**	0,442	1,27E-08	**157**	0,119	1,33E-02	**257**	0,249	2,80E-06	**357**	0,149	1,98E-03
**58**	0,208	5,22E-05	**158**	0,071	1,04E-01	**258**	0,226	1,03E-05	**358**	0,461	1,34E-09
**59**	0,309	3,98E-02	**159**	0,178	5,23E-04	**259**	0,365	1,06E-05	**359**	0,353	5,73E-03
**60**	0,260	1,70E-06	**160**	0,229	6,70E-06	**260**	0,284	1,00E-07	**360**	0,258	1,60E-06
**61**	0,209	3,78E-05	**161**	0,322	9,19E-03	**261**	0,230	6,40E-06	**361**	0,303	3,03E-02
**62**	0,266	8,00E-07	**162**	0,138	3,97E-03	**262**	0,465	6,15E-12	**362**	0,107	2,14E-02
**63**	0,417	1,37E-09	**163**	0,243	1,15E-05	**263**	0,310	5,23E-02	**363**	0,160	1,56E-03
**64**	0,351	9,46E-05	**164**	0,193	2,34E-04	**264**	0,288	2,02E-01	**364**	0,092	4,01E-02
**65**	0,189	1,54E-04	**165**	0,200	7,14E-05	**265**	0,354	7,29E-04	**365**	0,008	4,42E-01
**66**	0,333	3,22E-02	**166**	0,337	9,93E-07	**266**	0,237	1,13E-05	**366**	0,113	1,89E-02
**67**	0,346	1,95E-03	**167**	0,173	8,09E-04	**267**	0,235	1,40E-06	**367**	0,224	1,85E-05
**68**	0,219	5,04E-05	**168**	0,256	2,00E-07	**268**	0,287	7,23E-02	**368**	0,291	1,00E-07
**69**	0,131	9,90E-03	**169**	0,331	4,54E-02	**269**	0,289	4,26E-01	**369**	0,389	2,96E-06
**70**	0,272	7,00E-07	**170**	0,155	1,42E-03	**270**	0,373	2,65E-05	**370**	0,263	2,10E-06
**71**	0,225	1,69E-05	**171**	0,279	4,00E-07	**271**	0,314	1,39E-01	**371**	0,185	2,44E-04
**72**	0,236	4,00E-06	**172**	0,180	1,76E-03	**272**	0,229	7,30E-06	**372**	0,229	1,43E-05
**73**	0,177	7,24E-04	**173**	0,334	6,62E-04	**273**	0,164	2,04E-03	**373**	0,208	4,73E-05
**74**	0,166	1,15E-03	**174**	0,418	2,28E-11	**274**	0,209	1,11E-04	**374**	0,222	2,35E-05
**75**	0,286	3,00E-07	**175**	0,284	1,00E-07	**275**	0,154	1,26E-03	**375**	0,260	1,00E-07
**76**	0,014	4,04E-01	**176**	0,440	1,12E-10	**276**	0,098	3,41E-02	**376**	0,209	9,50E-05
**77**	0,121	1,05E-02	**177**	0,340	2,01E-03	**277**	0,196	3,82E-04	**377**	0,238	4,40E-06
**78**	0,130	9,71E-03	**178**	0,453	3,66E-10	**278**	0,137	6,42E-03	**378**	0,318	5,97E-03
**79**	0,197	1,11E-04	**179**	0,488	7,57E-15	**279**	0,152	2,69E-03	**379**	0,315	5,56E-03
**80**	0,142	5,45E-03	**180**	0,315	4,43E-02	**280**	0,119	1,62E-02	**380**	0,380	5,24E-05
**81**	0,399	3,28E-07	**181**	0,255	8,00E-07	**281**	0,087	4,68E-02	**381**	0,239	3,10E-06
**82**	0,266	3,00E-07	**182**	0,381	2,24E-06	**282**	0,258	1,30E-06	**382**	0,361	4,93E-04

ROI	h2	p	ROI	h2	p	ROI	h2	p	ROI	h2	p
**83**	0,395	2,39E-05	**183**	0,282	1,00E-07	**283**	0,198	2,01E-04	**383**	0,418	5,51E-08
**84**	0,345	5,04E-02	**184**	0,391	8,05E-08	**284**	0,121	1,12E-02	**384**	0,417	3,01E-06
**85**	0,305	1,00E-07	**185**	0,185	4,58E-04	**285**	0,279	1,00E-07	**385**	0,399	5,82E-07
**86**	0,090	4,08E-02	**186**	0,222	3,31E-05	**286**	0,376	1,82E-05	**386**	0,416	8,83E-08
**87**	0,182	3,42E-04	**187**	0,277	7,00E-07	**287**	0,254	2,90E-06	**387**	0,266	4,00E-07
**88**	0,137	6,42E-03	**188**	0,416	2,65E-07	**288**	0,340	6,16E-03	**388**	0,310	1,64E-02
**89**	0,376	5,08E-05	**189**	0,378	2,33E-05	**289**	0,300	2,73E-01	**389**	0,337	2,31E-03
**90**	0,224	5,17E-05	**190**	0,390	1,15E-05	**290**	0,220	7,36E-05	**390**	0,259	7,00E-07
**91**	0,174	7,52E-04	**191**	0,256	8,00E-07	**291**	0,231	1,81E-05	**391**	0,359	4,38E-05
**92**	0,177	3,90E-04	**192**	0,180	8,31E-04	**292**	0,149	3,17E-03	**392**	0,373	2,90E-05
**93**	0,238	7,50E-06	**193**	0,324	2,90E-02	**293**	0,179	3,26E-04	**393**	0,385	2,28E-04
**94**	0,307	1,19E-02	**194**	0,262	7,00E-07	**294**	0,195	3,27E-04	**394**	0,226	1,42E-05
**95**	0,221	2,22E-05	**195**	0,126	1,15E-02	**295**	0,123	1,66E-02	**395**	0,304	1,23E-01
**96**	0,252	3,60E-06	**196**	0,228	1,03E-05	**296**	0,195	1,75E-04	**396**	0,143	4,55E-03
**97**	0,404	1,42E-08	**197**	0,313	1,59E-01	**297**	0,183	2,22E-04	**397**	0,264	1,00E-07
**98**	0,364	1,23E-04	**198**	0,323	6,79E-03	**298**	0,197	2,45E-04	**398**	0,396	3,84E-07
**99**	0,126	8,51E-03	**199**	0,199	3,75E-04	**299**	0,358	1,71E-04	**399**	0,190	2,19E-04
**100**	0,434	8,36E-08	**200**	0,241	4,70E-06	**300**	0,116	1,19E-02	**400**	0,201	5,57E-05

Table S1. Heritability of cortical thickness in each parcel. Heritability of cortical thickness in each parcel, controlling for global thickness. Parcel numbers refer to the Schaefer 400, 7 networks solution (*25*).

**Gradient 1**			
T1w/T2w	Correlation with G1	T1w/T2w	Correlation with G1
Layer 1	-0.34, p<0.000001	Layer 7	-0.42, p<0.000001
Layer 2	-0.40, p<0.000001	Layer 8	-0.43, p<0.000001
Layer 3	-0.40, p<0.000001	Layer 9	-0.43, p<0.000001
Layer 4	-0.38, p<0.000001	Layer 10	-0.44, p<0.000001
Layer 5	-0.39, p<0.000001	Layer 11	-0.43, p<0.000001
Layer 6	-0.40, p<0.000001	Layer 12	-0.43, p<0.000001
**Gradient 2**			
T1w/T2w	Correlation with G2	T1w/T2w	Correlation with G2
Layer 1	0.64, p<0.000001	Layer 7	-0.05, p>ns
Layer 2	0.40, p<0.000001	Layer 8	-0.04, p>ns
Layer 3	0.12, p<0.02	Layer 9	-0.03, p>ns
Layer 4	-0.01, p>ns	Layer 10	-0.03, p>ns
Layer 5	-0.05, p>ns	Layer 11	-0.02, p>ns
Layer 6	-0.05, p>ns	Layer 12	0.00, p>ns

Table S2. Correlation between layer-dependent T1w/T2w and covariance gradients. Correlation between layer-based T1w/T2w and the first two gradient of thickness covariance (G1 and G2, please see Fig 1).

SUPPLEMENTARY FIGURES

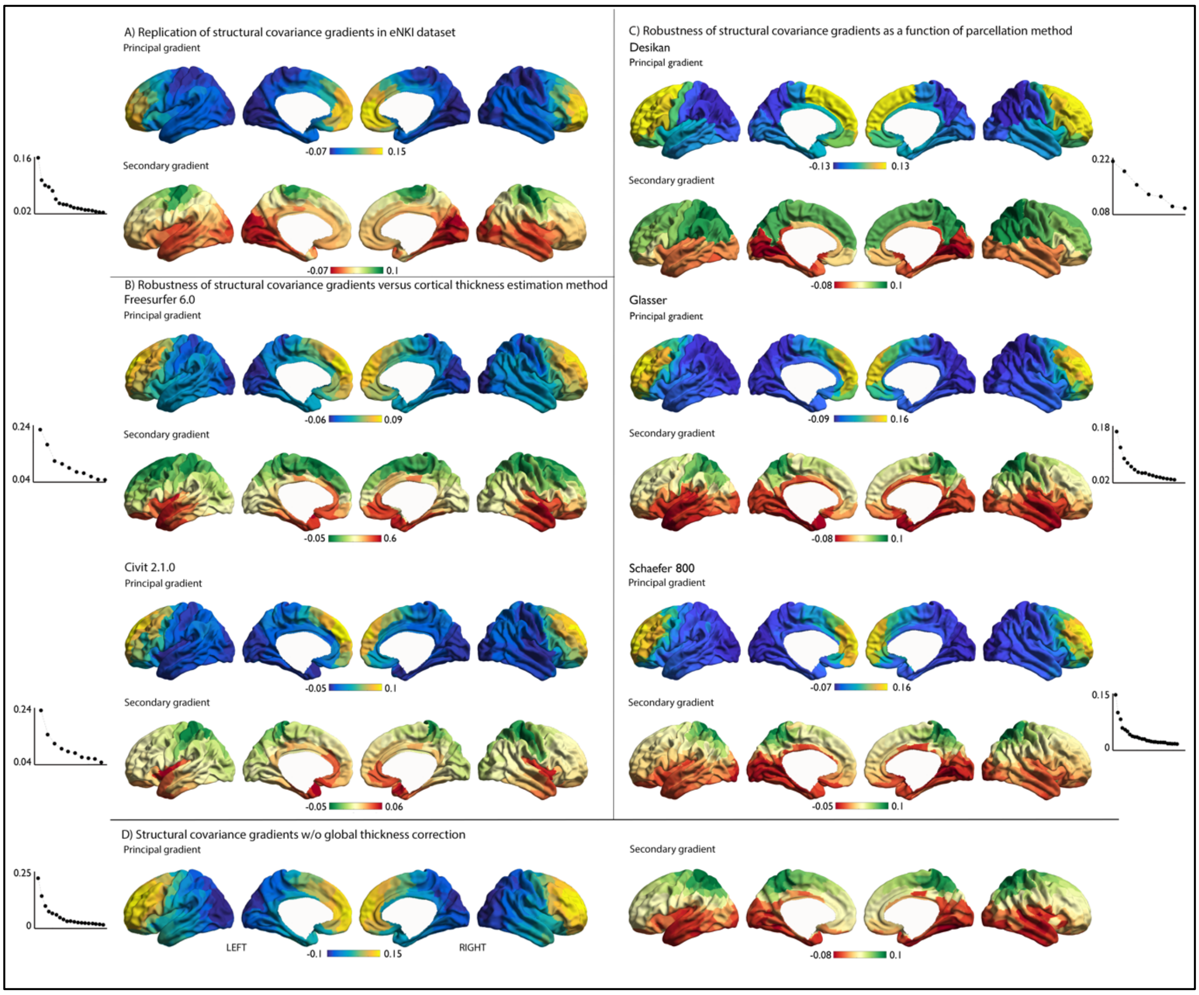

Fig S1. Replication of structural covariance gradients. A) Replication of the first two gradients in the eNKI dataset, using the Schaefer 400 parcellation; **B**) Cortical thickness estimation in HCP sample based on Freesurfer 6.0 standard pipeline and CIVIT 2.1.0. standard pipeline; **C**) Cortical thickness parcellated using the Desikan-Killiany atlas; the Glasser atlas; and the Schaefer 800 atlas; **D**) Cortical thickness covariance without global distance correction.

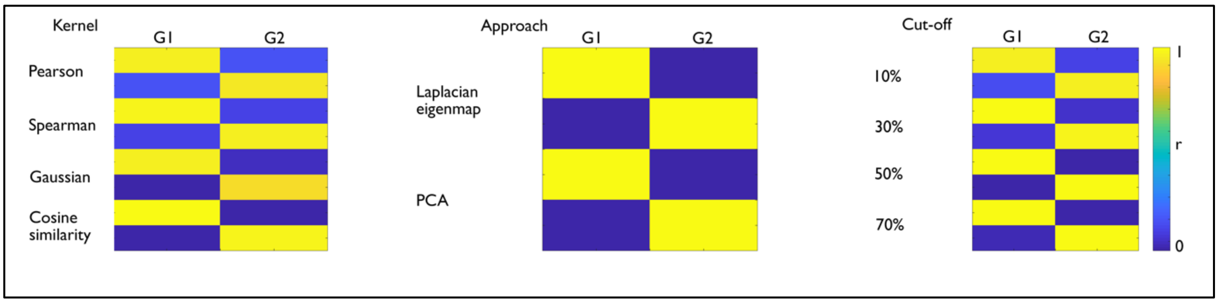

Fig S2. Robustness of structural covariance gradients as a function of gradient construction. Correlation of principal and secondary gradient in structural covariance reported in F1 with gradients produced with other kernel/approach/cut-off values. *Left*: kernel variation: Pearson, Spearman, Gaussian and Cosine Similarity kernels; *Middle*: approach: Laplacian eigenmaps; PCA; *Right*: cut-off (10, 30, 50, 70% percentile cut-off of covariance matrix).

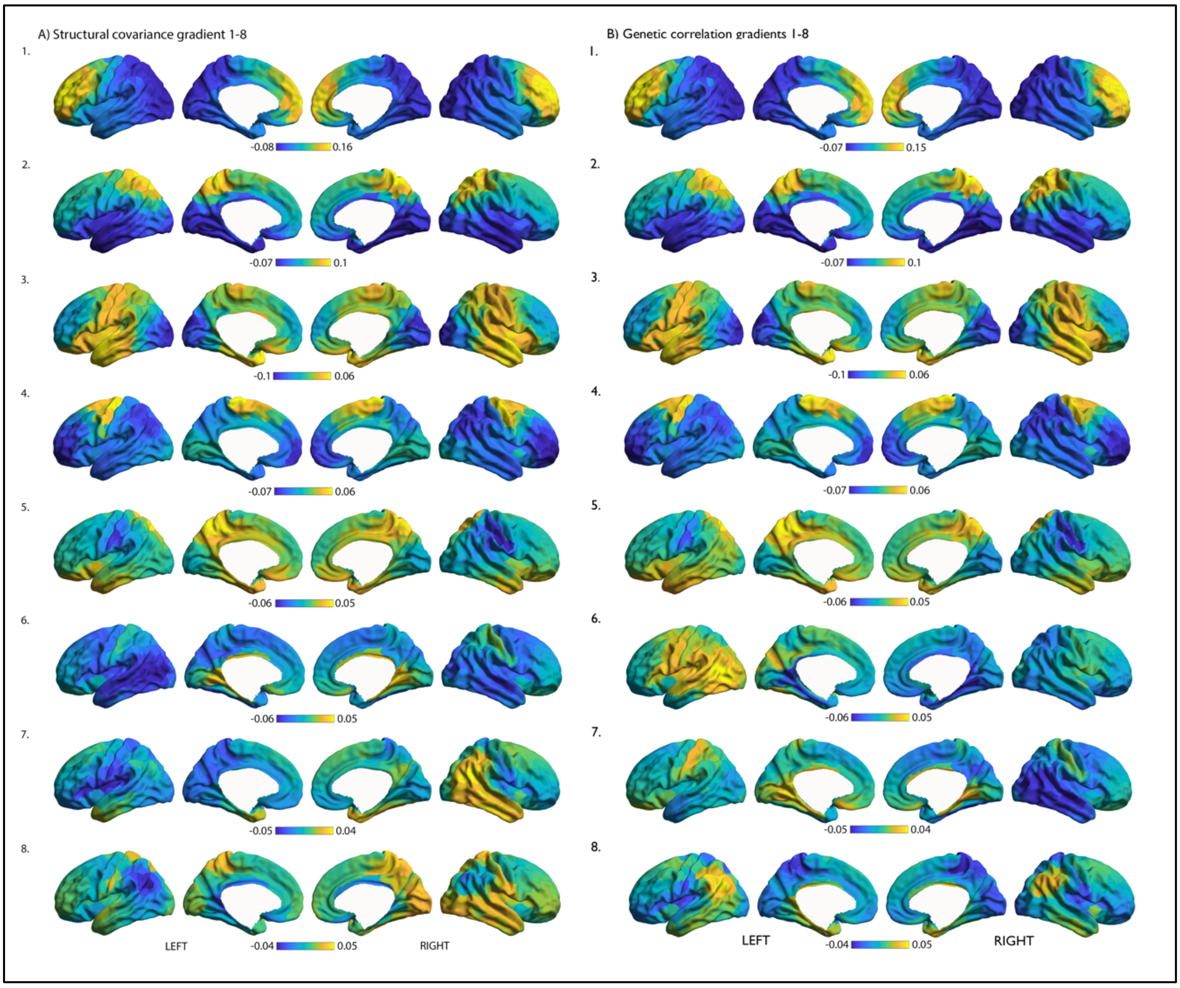

Fig S3. Gradients of structural covariance and genetic correlation. The first eight gradients of structural covariance of thickness (A) and genetic correlation (B), all in same color scheme.

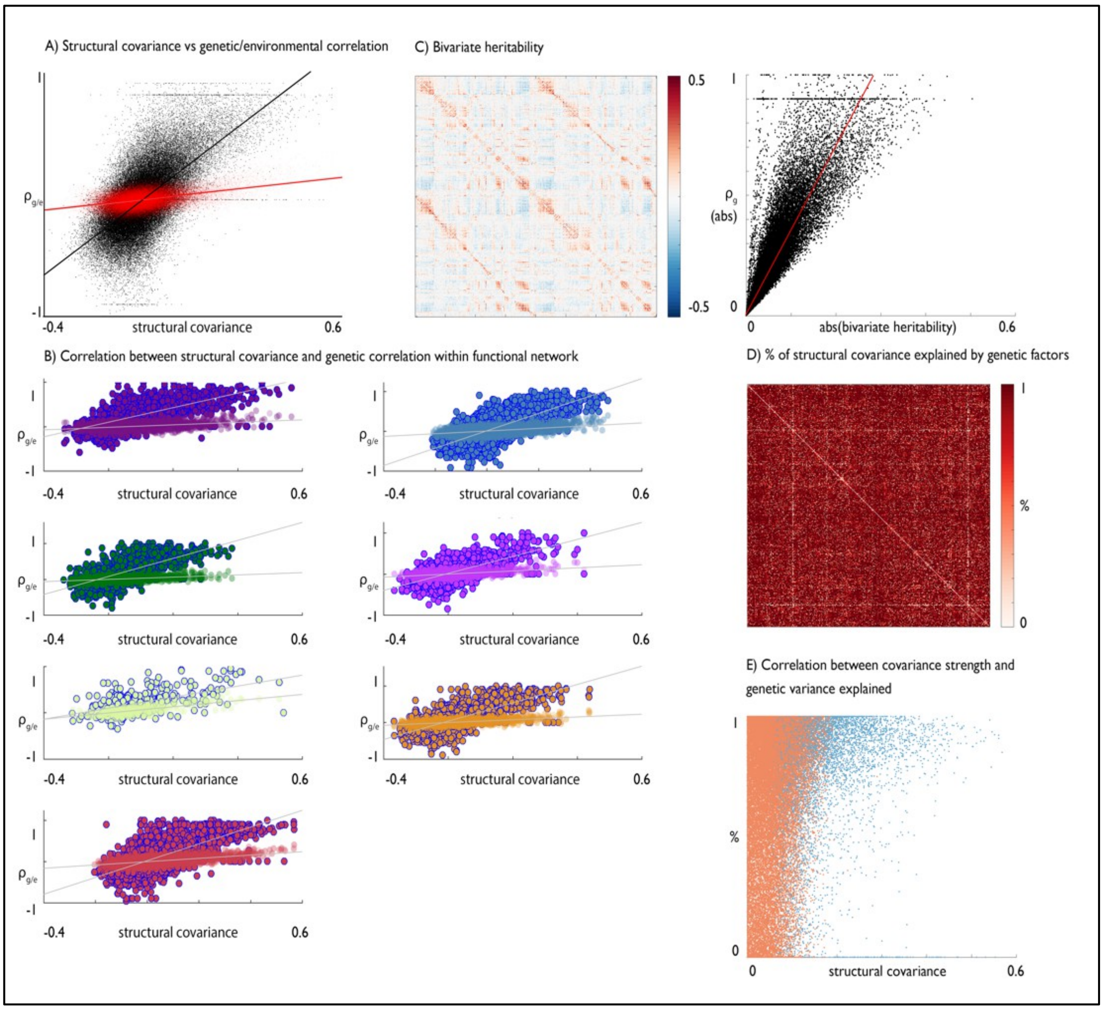

Fig S4. Structural covariance of thickness and its genetic and environmental components. **A**) Whole brain correlation between covariance and genetic correlation (black) and environmental correlation (red). **B**) Correlation between covariance and genetic correlation (blue outline) and environmental correlation (no outline) within each functional community (*25*); **C**) Bivariate heritability; **D**) Percentage of phenotypic correlation explained by genetic factors; **E**) Association between structural covariance and percentage of phenotypic correlation explained by genetic factors, covariance pairs part of the row-wise top 10% used for covariance are highlighted in blue.

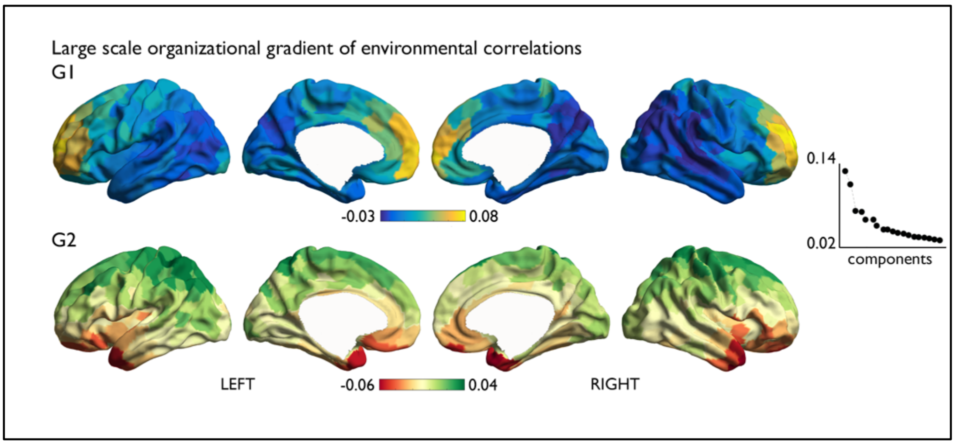

Fig S5. Large-scale organizational gradients of environmental correlations of thickness. Performing non-linear matrix decomposition methods on the environmental correlation of thickness.

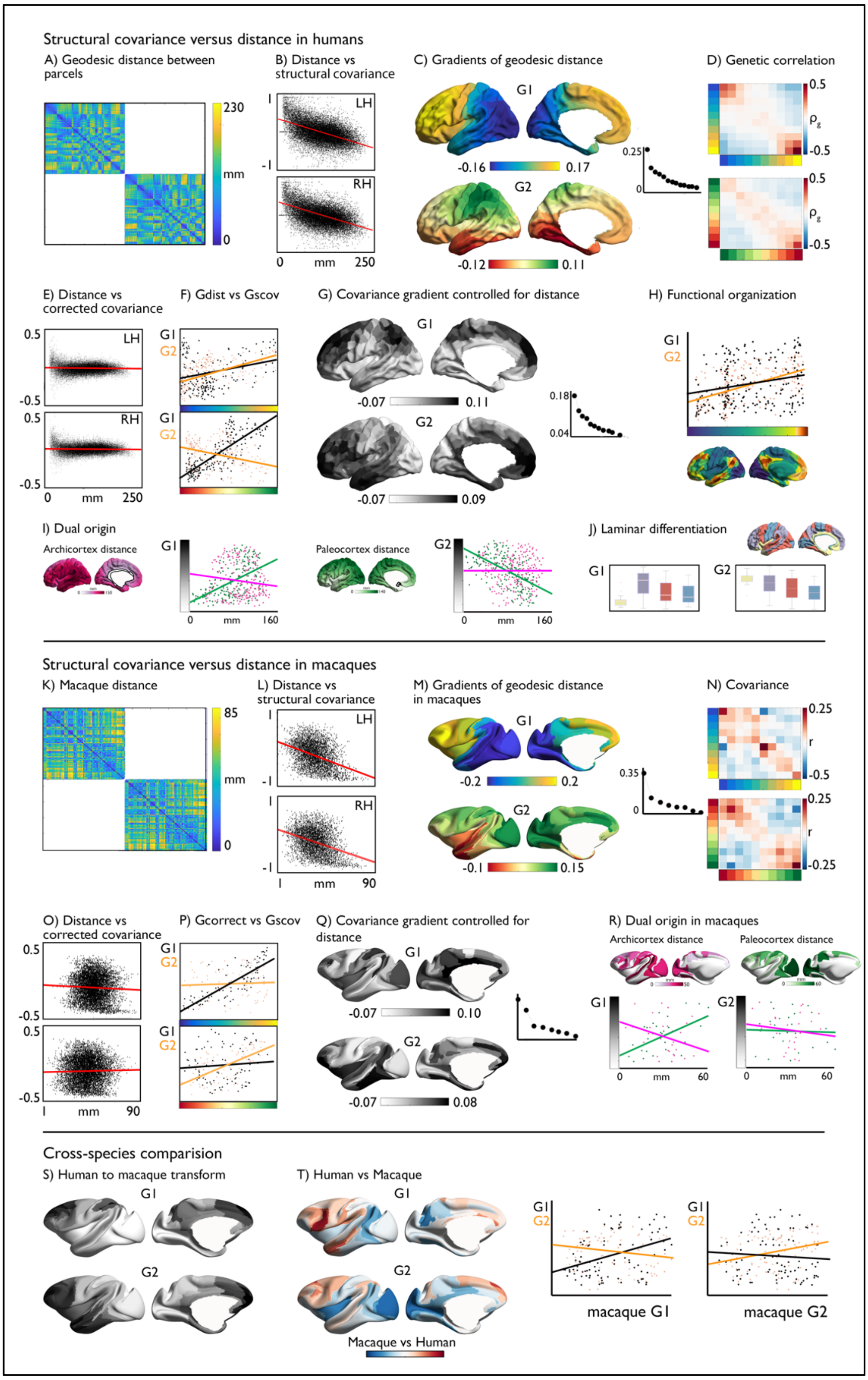

Fig S6: Controlling for distance in structural covariance. **A**) Geodesic distance matrix of ipsilateral 400 Schaefer parcels (*25*); **B**) Correlation between geodesic distance and structural covariance between parcels; **C**) Principal and secondary gradient of geodesic distance; **D**) Genetic correlation as a function of the binned geodesic distance gradients; **E**) Correlation between geodesic distance and distance-regressed structural covariance; **F**) Distance corrected gradient versus covariance gradients reported in Fig 1; **G**) Covariance gradients while controlling for geodesic distance; **H**) Distance-corrected gradients versus large-scale functional gradient (*28*); **I**) Relation of binned-gradients to distance from archi- and paleocortex; **J**) Distance-corrected gradients versus laminar differentiation; **K**) Geodesic distance in Markov parcellation in macaques (*32*); **L**) Correlation between geodesic distance and structural covariance between parcels in macaques; **M**) Principal and secondary gradient of geodesic distance in macaques; **N**) Covariance as a function of the binned geodesic distance gradients in macaques;

O) Correlation between geodesic distance and distance-regressed structural covariance in macaques; **P**) Distance corrected gradient versus macaque covariance gradients reported in Fig 3; **Q**) Macaque covariance gradients while controlling for geodesic distance; **R**) Relation of binned-gradients to distance from archi- and paleocortex in macaques; **S**) Cross-species comparison transformed human gradient to macaque surface; **T**) Human versus macaque distance corrected gradient; *Right:* Scatter plot of both macaque (x-axis) and human (y-axis) distance corrected covariance gradients.

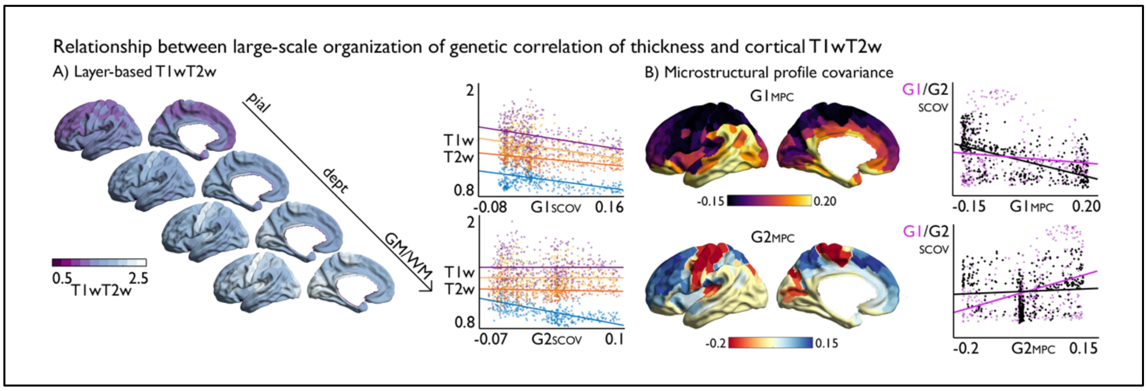

Fig S7. Link between organization of macro-scale organization of thickness and microstructure. A) Relationship between large-scale organization of genetic correlation of thickness and cortical T1w/T2w; i. T1w/T2w values of equidistant layers between the pial and GM/WM surface and the correlation with the principal and secondary gradient (G1SCOV and G2SCOV) of macro-scale organization of thickness. For visualization purposes only the first (blue), fourth(orange), seventh (yellow), tenth (purple) of 12 probed layers are reported; **B)** Principal and secondary gradient of microstructure profile covariance (MPC) and the relationship between MPC gradients and G1SCOV and G2SCOV.

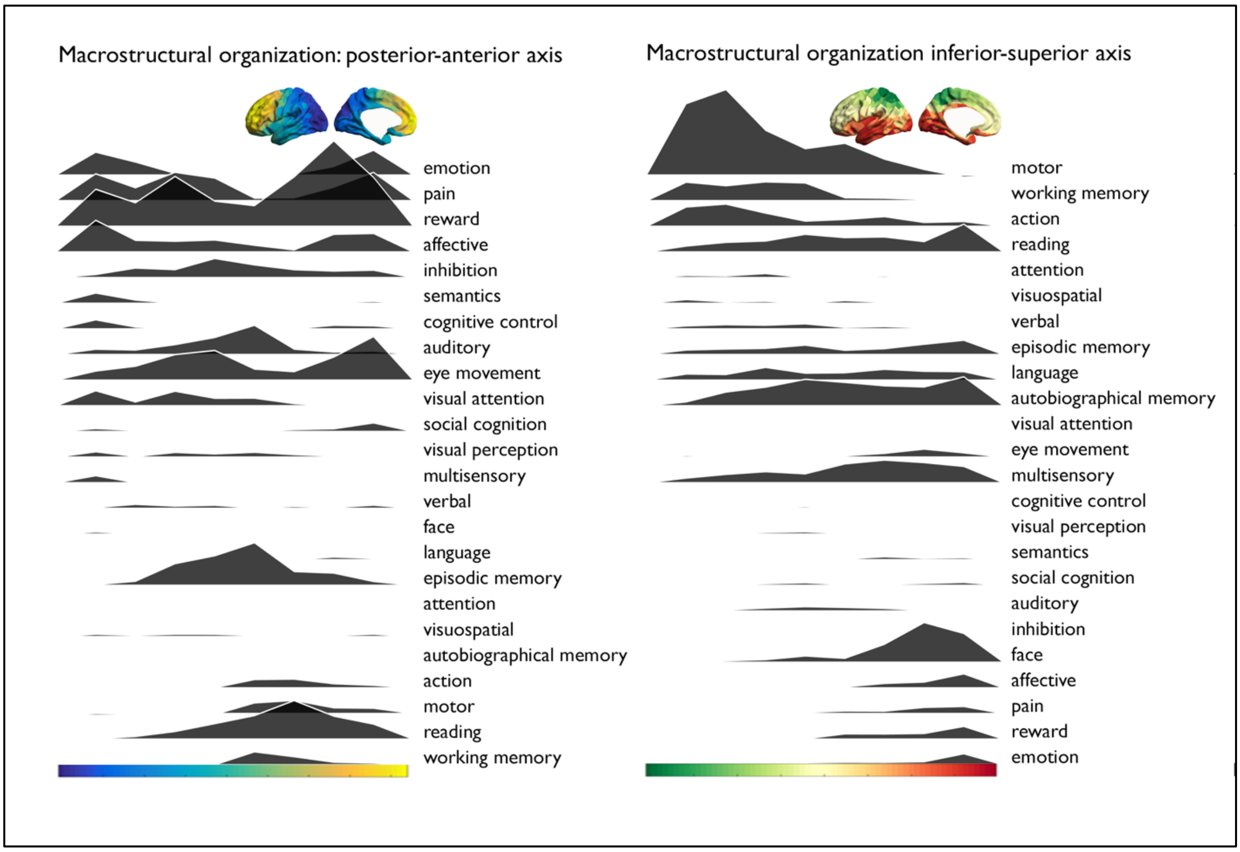

Fig S8. Neurosynth functional meta-analysis. Meta-analysis maps for diverse cognitive terms were obtained from Neurosynth similar to Margulies et al. (*28*). We calculated parcel-wise z-statistics, capturing node-term associations, and calculated the center of gravity of each term along the poster-anterior and inferior-superior gradients. The plots depict the average z-score within binned (20-bins) gradient layer of meta-analysis maps.

REFERENCES AND NOTES

1. J. M. Huntenburg, P.-L. Bazin, D. S. Margulies, Large-scale gradients in human cortical organization. *Trends Cogn. Sci.* **22**, 21–31 (2018).

2. F. Sanides, *Die Archtektonik des Menschlichen Stirnhirns* (Springer, 1962).

3. D. N. Pandya, M. Petrides, B. Seltzer, B. P. Cipolloni, *Cerebral Cortex: Architecture, Connections, and the Dual Origin Concept* (Oxford Press, 2015).

4. R. A. Dart, The Dual Structure of the Neopallium: Its History and Significance. *J. Anat.* **69**, 3–19 (1934).

5. A. A. Abbie, Cortical lamination in the monotremata. *J Comp Neurol* **72**, 429–467 (1940).

6. M. M. Mesulam, From sensation to cognition. *Brain* **121** (Pt 6), 1013–1052 (1998).

7. C. Murphy, E. Jefferies, S.-A. Rueschemeyer, M. Sormaz, H.-T. Wang, D. S. Margulies, J. Smallwood, Distant from input: Evidence of regions within the default mode network supporting perceptually-decoupled and conceptually-guided cognition. *Neuroimage* **171**, 393–401 (2018).

8. H. Braak, E. Braak, Alzheimer's disease affects limbic nuclei of the thalamus. *Acta Neuropathol.* **81**, 261–268 (1991).

9. S.-J. Hong, R. V. de Wael, R. A. I. Bethlehem, S. Lariviere, C. Paquola, S. L. Valk, M. P. Milham, A. D. Martino, D. S. Margulies, J. Smallwood, B. C. Bernhardt, Atypical functional connectome hierarchy in autism. *Nat. Commun.* **10**, 1022 (2019).

10. D. J. Cahalane, C. J. Charvet, B. L. Finlay, Systematic, balancing gradients in neuron density and number across the primate isocortex. *Front Neuroanat* **6**, 28 (2012).

11. K. Wagstyl, L. Ronan, I. M. Goodyer, P. C. Fletcher, Cortical thickness gradients in structural hierarchies. *Neuroimage* **111**, 241–250 (2015).

12. J. E. Schmitt, R. K. Lenroot, S. E. Ordaz, G. L. Wallace, J. P. Lerch, A. C. Evans, E. C. Prom, K. S. Kendler, M. C. Neale, J. N. Giedd, Variance decomposition of MRI-based covariance maps using genetically informative samples and structural equation modeling. *Neuroimage* **47**, 56–64 (2009).

13. A. F. Alexander-Bloch, S. R. Mathias, P. T. Fox, R. L. Olvera, H. H. H. Göring, R. Duggirala, J. E. Curran, J. Blangero, D. C. Glahn, Human cortical thickness organized into genetically-determined communities across spatial resolutions. *Cereb. Cortex* **29**, 106–118 (2019).

14. C.-H. Chen, M. Fiecas, E. D. Gutiérrez, M. S. Panizzon, L. T. Eyler, E. Vuoksimaa, W. K. Thompson, C. Fennema-Notestine, D. J. Hagler Jr., T. L. Jernigan, M. C. Neale, C. E. Franz, M. J. Lyons, B. Fischl, M. T. Tsuang, A. M. Dale, W. S. Kremen, Genetic topography of brain morphology. *Proc. Natl. Acad. Sci. U.S.A.* **110**, 17089–17094 (2013).

15. A. Raznahan, J. P. Lerch, N. Lee, D. Greenstein, G. L. Wallace, M. Stockman, L. Clasen, P. W. Shaw, J. N. Giedd, Patterns of coordinated anatomical change in human cortical development: A longitudinal neuroimaging study of maturational coupling. *Neuron* **72**, 873–884 (2011).

16. A. R. Docherty, C. K. Sawyers, M. S. Panizzon, M. C. Neale, L. T. Eyler, C. Fennema-Notestine, C. E. Franz, C.-H. Chen, L. K. McEvoy, B. Verhulst, M. T. Tsuang, W. S. Kremen, Genetic network properties of the human cortex based on regional thickness and surface area measures. *Front. Hum. Neurosci.* **9**, 440 (2015).

17. J. P. Lerch, K. Worsley, W. P. Shaw, D. K. Greenstein, R. K. Lenroot, J. Giedd, A. C. Evans, Mapping anatomical correlations across cerebral cortex (MACACC) using cortical thickness from MRI. *Neuroimage* **31**, 993–1003 (2006).

18. G. Gong, Y. He, Z. J. Chen, A. C. Evans, Convergence and divergence of thickness correlations with diffusion connections across the human cerebral cortex. *Neuroimage* **59**, 1239–1248 (2012).

19. A. Alexander-Bloch, J. N. Giedd, E. Bullmore, Imaging structural co-variance between human brain regions. *Nat. Rev. Neurosci.* **14**, 322–336 (2013).

20. R. Romero-Garcia, K. J. Whitaker, F. Váša, J. Seidlitz, M. Shinn, P. Fonagy, R. J. Dolan, P. B. Jones, I. M. Goodyer; NSPN Consortium, E. T. Bullmore, P. E. Vértes, Structural covariance

networks are coupled to expression of genes enriched in supragranular layers of the human cortex. *Neuroimage* **171**, 256–267 (2018).

21. Y. Yee, D. J. Fernandes, L. French, J. Ellegood, L. S. Cahill, D. A. Vousden, L. S. Noakes, J. Scholz, M. C van Eede, B. J. Nieman, J. G. Sled, J. P. Lerch, Structural covariance of brain region volumes is associated with both structural connectivity and transcriptomic similarity. *Neuroimage* **179**, 357–372 (2018).

22. M. J. Hawrylycz, E. S. Lein, A. L. Guillozet-Bongaarts, E. H. Shen, L. Ng, J. A. Miller, L. N. van de Lagemaat, K. A. Smith, A. Ebbert, Z. L. Riley, C. Abajian, C. F. Beckmann, A. Bernard, D. Bertagnolli, A. F. Boe, P. M. Cartagena, M. M. Chakravarty, M. Chapin, J. Chong, R. A. Dalley, B. D. Daly, C. Dang, S. Datta, N. Dee, T. A. Dolbeare, V. Faber, D. Feng, D. R. Fowler, J. Goldy, B. W. Gregor, Z. Haradon, D. R. Haynor, J. G. Hohmann, S. Horvath, R. E. Howard, A. Jeromin, J. M. Jochim, M. Kinnunen, C. Lau, E. T. Lazarz, C. Lee, T. A. Lemon, L. Li, Y. Li, J. A. Morris, C. C. Overly, P. D. Parker, S. E. Parry, M. Reding, J. J. Royall, J. Schulkin, P. A. Sequeira, C. R. Slaughterbeck, S. C. Smith, A. J. Sodt, S. M. Sunkin, B. E. Swanson, M. P. Vawter, D. Williams, P. Wohnoutka, H. R. Zielke, D. H. Geschwind, P. R. Hof, S. M. Smith, C. Koch, S. G. N. Grant, A. R. Jones, An anatomically comprehensive atlas of the adult human brain transcriptome. *Nature* **489**, 391–399 (2012).

23. B. T. T. Yeo, F. M. Krienen, J. Sepulcre, M. R. Sabuncu, D. Lashkari, M. Hollinshead, J. L. Roffman, J. W. Smoller, L. Zöllei, J. R. Polimeni, B. Fischl, H. Liu, R. L. Buckner, The organization of the human cerebral cortex estimated by intrinsic functional connectivity. *J. Neurophysiol.* **106**, 1125–1165 (2011).

24. M. P. Milham, L. Ai, B. Koo, T. Xu, C. Amiez, F. Balezeau, M. G. Baxter, E. L. A. Blezer, T. Brochier, A. Chen, P. L. Croxson, C. G. Damatac, S. Dehaene, S. Everling, D. A. Fair, L. Fleysher, W. Freiwald, S. Froudist-Walsh, T. D. Griffiths, C. Guedj, F. Hadj-Bouziane, S. B. Hamed, N. Harel, B. Hiba, B. Jarraya, B. Jung, S. Kastner, P. C. Klink, S. C. Kwok, K. N. Laland, D. A. Leopold, P. Lindenfors, R. B. Mars, R. S. Menon, A. Messinger, M. Meunier, K. Mok, J. H. Morrison, J. Nacef, J. Nagy, M. O. Rios, C. I. Petkov, M. Pinsk, C. Poirier, E. Procyk, R. Rajimehr, S. M. Reader, P. R. Roelfsema, D. A. Rudko, M. F. S. Rushworth, B. E. Russ, J. Sallet, M. C.

Schmid, C. M. Schwiedrzik, J. Seidlitz, J. Sein, A. Shmuel, E. L. Sullivan, L. Ungerleider, A. Thiele, O. S. Todorov, D. Tsao, Z. Wang, C. R. E. Wilson, E. Yacoub, F. Q. Ye, W. Zarco, Y.-d. Zhou, D. S. Margulies, C. E. Schroeder, An Open resource for non-human primate imaging. *Neuron* **100**, 61–74.e2 (2018).

25. A. Schaefer, R. Kong, E. M. Gordon, T. O. Laumann, X.-N. Zuo, A. J. Holmes, S. B. Eickhoff, B. T. T. Yeo, Local-global parcellation of the human cerebral cortex from intrinsic functional connectivity MRI. *Cereb. Cortex* **28**, 3095–3114 (2018).

26. A. Alexander-Bloch, A. Raznahan, E. Bullmore, J. Giedd, The convergence of maturational change and structural covariance in human cortical networks. *J. Neurosci.* **33**, 2889–2899 (2013).

27. C. Paquola, R. V. De Wael, K. Wagstyl, R. A. I. Bethlehem, S.-J. Hong, J. Seidlitz, E. T. Bullmore, A. C. Evans, B. Misic, D. S. Margulies, J. Smallwood, B. C. Bernhardt, Microstructural and functional gradients are increasingly dissociated in transmodal cortices. *PLOS Biol.* **17**, e3000284 (2019).

28. D. S. Margulies, S. S. Ghosh, A. Goulas, M. Falkiewicz, J. M. Huntenburg, G. Langs, G. Bezgin, S. B. Eickhoff, F. X. Castellanos, M. Petrides, E. Jefferies, J. Smallwood, Situating the default-mode network along a principal gradient of macroscale cortical organization. *Proc. Natl. Acad. Sci. U.S.A.* **113**, 12574–12579 (2016).

29. R. Vos De Wael, O. Benkarim, C. Paquola, S. Lariviere, J. Royer, S. Tavakol, T. Xu, S.-J. Hong, G. Langs, S. Valk, B. Misic, M. Milham, D. Margulies, J. Smallwood, B. C. Bernhardt, BrainSpace: A toolbox for the analysis of macroscale gradients in neuroimaging and connectomics datasets. *Commun. Biol.* **3**, 103 (2020).

30. R. S. Desikan, F. Ségonne, B. Fischl, B. T. Quinn, B. C. Dickerson, D. Blacker, R. L. Buckner, A. M. Dale, R. P. Maguire, B. T. Hyman, M. S. Albert, R. J. Killiany, An automated labeling system for subdividing the human cerebral cortex on MRI scans into gyral based regions of interest. *Neuroimage* **31**, 968–980 (2006).

31. M. F. Glasser, T. S. Coalson, E. C. Robinson, C. D. Hacker, J. Harwell, E. Yacoub, K. Ugurbil, J. Andersson, C. F. Beckmann, M. Jenkinson, S. M. Smith, D. C. Van Essen, A multi-modal parcellation of human cerebral cortex. *Nature* **536**, 171–178 (2016).

32. N. T. Markov, M. M. Ercsey-Ravasz, A. R. R. Gomes, C. Lamy, L. Magrou, J. Vezoli, P. Misery, A. Falchier, R. Quilodran, M. A. Gariel, J. Sallet, R. Gamanut, C. Huissoud, S. Clavagnier, P. Giroud, D Sappey-Marinier, P. Barone, C. Dehay, Z. Toroczkai, K. Knoblauch, D. C. Van Essen, H. Kennedy, A weighted and directed interareal connectivity matrix for macaque cerebral cortex. *Cereb. Cortex* **24**, 17–36 (2014).

33. T. Xu, K.-H. Nenning, E. Schwartz, S.-J. Hong, J. T. Vogelstein, D. A. Fair, C. E. Schroeder, D. S. Margulies, J. Smallwood, M. P. Milham, G. Langs, Cross-species functional alignment reveals evolutionary hierarchy within the connectome. *bioRxiv*, 692616 (2019).

34. A. Goulas, D. S. Margulies, G. Bezgin, C. C. Hilgetag, The architecture of mammalian cortical connectomes in light of the theory of the dual origin of the cerebral cortex. *Cortex* **118**, 244–261 (2019).

35. B. Aslan, G. Zech, Statistical energy as a tool for binning-free, multivariate goodness-of-fit tests, two-sample comparison and unfolding. *Nucl. Instrum. Methods Phys. Res. Section A.* **537**, 626–636 (2005).

36. C. J. Charvet, D. J. Cahalane, B. L. Finlay, Systematic, cross-cortex variation in neuron numbers in rodents and primates. *Cereb. Cortex* **25**, 147–160 (2014).

37. C. J. Charvet, B. L. Finlay, Evo-devo and the primate isocortex: The central organizing role of intrinsic gradients of neurogenesis. *Brain Behav. Evol.* **84**, 81–92 (2014).

38. G. N. Elston, Pyramidal cells of the frontal lobe: All the more spinous to think with. *J. Neurosci.* **20**, RC95 (2000).

39. D. J. Cahalane, C. J. Charvet, B. L. Finlay, Modeling local and cross-species neuron number variations in the cerebral cortex as arising from a common mechanism. *Proc. Natl. Acad. Sci. U.S.A.* **111**, 17642–17647 (2014).

40. A. Fornito, A. Arnatkevičiūtė, B. D. Fulcher, Bridging the gap between connectome and transcriptome. *Trends Cogn. Sci.* **23**, 34–50 (2019).

41. D. N. Pandya, E. H. Yeterian, in *Association and auditory cortices*, A. Peters, E. G. Jones, Eds. (Springer Science Business Media, New York, 1985), pp. 3–61.

42. H. Barbas, D. N. Pandya, Architecture and intrinsic connections of the prefrontal cortex in the rhesus monkey. *J. Comp. Neurol.* **286**, 353–375 (1989).

43. R. G. Giaccio, The dual origin hypothesis: An evolutionary brain-behavior framework for analyzing psychiatric disorders. *Neurosci. Biobehav. Rev.* **30**, 526–550 (2006).

44. D. Saur, B. W. Kreher, S. Schnell, D. Kümmerer, P. Kellmeyer, M.-S. Vry, R. Umarova, M. Musso, V. Glauche, S. Abel, W. Huber, M. Rijntjes, J. Hennig, C. Weiller, Ventral and dorsal pathways for language. *Proc. Natl. Acad. Sci. U.S.A.* **105**, 18035–18040 (2008).

45. P. Rakic, Neurogenesis in adult primate neocortex: An evaluation of the evidence. *Nat. Rev. Neurosci.* **3**, 65–71 (2002).

46. P. Gunz, A. K. Tilot, K. Wittfeld, A. Teumer, C. Y. Shapland, T. G. M. van Erp, M. Dannemann, B. Vernot, S. Neubauer, T. Guadalupe, G. Fernández, H. G. Brunner, W. Enard, J. Fallon, N. Hosten, U. Völker, A. Profico, F. D. Vincenzo, G. Manzi, J. Kelso, B. S. Pourcain, J.-J. Hublin, B. Franke, S. Pääbo, F. Macciardi, H. J. Grabe, S. E. Fisher, Neandertal introgression sheds light on modern human endocranial globularity. *Curr. Biol.* **29**, 895 (2019).

47. O. Vogt, Die myeloarchitektonik des isocrotex parietalis. *J. Psychol. Neurol.* **18**, 379–390 (1911).

48. C. Paquola, R. A. I. Bethlehem, J. Seidlitz, K. Wagstyl, R. Romero-Garcia, K. J. Whitaker, R. V. de Wael, G. B. Williams; NSPN Consortium, P. E. Vértes, D. S. Margulies, B. Bernhardt, E. T. Bullmore, A moment of change: Shifts in myeloarchitecture characterise adolescent development of cortical gradients. *eLife*, e50482 (2020).

49. D. J. Felleman, D. C. Van Essen, Distributed hierarchical processing in the primate cerebral cortex. *Cereb. Cortex* **1**, 1–47 (1991).

50. E. Bullmore, O. Sporns, The economy of brain network organization. *Nat. Rev. Neurosci.* **13**, 336–349 (2012).

51. D. D. M. O'Leary, S.-J. Chou, S. Sahara, Area patterning of the mammalian cortex. *Neuron* **56**, 252–269 (2007).

52. P. E. Vértes, A. F. Alexander-Bloch, N. Gogtay, J. N. Giedd, J. L. Rapoport, E. T. Bullmore, Simple models of human brain functional networks. *Proc. Natl. Acad. Sci. U.S.A.* **109**, 5868–5873 (2012).

53. J. D. Power, K. A. Barnes, A. Z. Snyder, B. L. Schlaggar, S. E. Petersen, Spurious but systematic correlations in functional connectivity MRI networks arise from subject motion. *Neuroimage* **59**, 2142–2154 (2012).

54. A. Fornito, A. Zalesky, M. Breakspear, Graph analysis of the human connectome: Promise, progress, and pitfalls. *Neuroimage* **80**, 426–444 (2013).

55. A. F. Alexander-Bloch, H. Shou, S. Liu, T. D. Satterthwaite, D. C. Glahn, R. T. Shinohara, S. N. Vandekar, A. Raznahan, On testing for spatial correspondence between maps of human brain structure and function. *Neuroimage* **178**, 540–551 (2018).

56. K. L. Grasby, N. Jahanshad, J. N. Painter, L. Colodro-Conde, J. Bralten, D. P. Hibar, P. A. Lind, F. Pizzagalli, C. R. K. Ching, M. A. B. McMahon, N. Shatokhina, L. C. P. Zsembik, S. I. Thomopoulos, A. H. Zhu, L. T. Strike, I. Agartz, S. Alhusaini, M. A. A. Almeida, D. Alnæs, I. K. Amlien, M. Andersson, T. Ard, N. J. Armstrong, A. Ashley-Koch, J. R. Atkins, M. Bernard, R. M. Brouwer, E. E. L. Buimer, R. Bülow, C. Bürger, D. M. Cannon, M. Chakravarty, Q. Chen, J. W. Cheung, B. Couvy-Duchesne, A. M. Dale, S. Dalvie, T. K. de Araujo, G. I. de Zubicaray, S. M. C. de Zwarte, A. den Braber, N. T. Doan, K. Dohm, S. Ehrlich, H.-R. Engelbrecht, S. Erk, C. C. Fan, I. O. Fedko, S. F. Foley, J. M. Ford, M. Fukunaga, M. E. Garrett, T. Ge, S. Giddaluru, A. L. Goldman, M. J. Green, N. A. Groenewold, D. Grotegerd, T. P. Gurholt, B. A. Gutman, N. K. Hansell, M. A. Harris, M. B. Harrison, C. C. Haswell, M. Hauser, S. Herms, D. J. Heslenfeld, N. F. Ho, D. Hoehn, P. Hoffmann, L. Holleran, M. Hoogman, J.-J. Hottenga, M. Ikeda, D. Janowitz, I. E. Jansen, T. Jia, C. Jockwitz, R. Kanai, S. Karama, D. Kasperaviciute, T. Kaufmann, S. Kelly, M. Kikuchi, M. Klein, M. Knapp, A. R. Knodt, B. Krämer, M. Lam, T. M. Lancaster, P. H. Lee, T. A. Lett, L. B. Lewis, I.

Lopes-Cendes, M. Luciano, F. Macciardi, A. F. Marquand, S. R. Mathias, T. R. Melzer, Y. Milaneschi, N. Mirza-Schreiber, J. C. V. Moreira, T. W. Mühleisen, B. Müller-Myhsok, P. Najt, S. Nakahara, K. Nho, L. M. O. Loohuis, D. P. Orfanos, J. F. Pearson, T. L. Pitcher, B. Pütz, Y. Quidé, A. Ragothaman, F. M. Rashid, W. R. Reay, R. Redlich, C. S. Reinbold, J. Repple, G. Richard, B. C. Riedel, S. L. Risacher, C. S. Rocha, N. R. Mota, L. Salminen, A. Saremi, A. J. Saykin, F. Schlag, L. Schmaal, P. R. Schofield, R. Secolin, C. Y. Shapland, L. Shen, J. Shin, E. Shumskaya, I. E. Sønderby, E. Sprooten, K. E. Tansey, A. Teumer, A. Thalamuthu, D. Tordesillas-Gutiérrez, J. A. Turner, A. Uhlmann, C. L. Vallerga, D. van der Meer, M. M. J. van Donkelaar, L. van Eijk, T. G. M. van Erp, N. E. M. van Haren, D. van Rooij, M.-J. van Tol, J. H. Veldink, E. Verhoef, E. Walton, M. Wang, Y. Wang, J. M. Wardlaw, W. Wen, L. T. Westlye, C. D. Whelan, S. H. Witt, K. Wittfeld, C. Wolf, T. Wolfers, J. Q. Wu, C. L. Yasuda, D. Zaremba, Z. Zhang, M. P. Zwiers, E. Artiges, A. A. Assareh, R. Ayesa-Arriola, A. Belger, C. L. Brandt, G. G. Brown, S. Cichon, J. E. Curran, G. E. Davies, F. Degenhardt, M. F. Dennis, B. Dietsche, S. Djurovic, C. P. Doherty, R. Espiritu, D. Garijo, Y. Gil, P. A. Gowland, R. C. Green, A. N. Häusler, W. Heindel, B.-C. Ho, W. U. Hoffmann, F. Holsboer, G. Homuth, N. Hosten, C. R. Jack Jr., M. H. Jang, A. Jansen, N. A. Kimbrel, K. Kolskår, S. Koops, A. Krug, K. O. Lim, J. J. Luykx, D. H. Mathalon, K. A. Mather, V. S. Mattay, S. Matthews, J. M. Van Son, S. C. McEwen, I. Melle, D. W. Morris, B. A. Mueller, M. Nauck, J. E. Nordvik, M. M. Nöthen, D. S. O'Leary, N. Opel, M.-L. P. Martinot, G. B. Pike, A. Preda, E. B. Quinlan, P. E. Rasser, V. Ratnakar, S. Reppermund, V. M. Steen, P. A. Tooney, F. R. Torres, D. J. Veltman, J. T. Voyvodic, R. Whelan, T. White, H. Yamamori, H. H. H. Adams, J. C. Bis, S. Debette, C. Decarli, M. Fornage, V. Gudnason, E. Hofer, M. A. Ikram, L. Launer, W. T. Longstreth, O. L. Lopez, B. Mazoyer, T. H. Mosley, G. V. Roshchupkin, C. L. Satizabal, R. Schmidt, S. Seshadri, Q. Yang; Alzheimer's Disease Neuroimaging Initiative; CHARGE Consortium; EPIGEN Consortium; IMAGEN Consortium; SYS Consortium; Parkinson's Progression Markers Initiative; M. K. M. Alvim, D. Ames, T. J. Anderson, O. A. Andreassen, A. Arias-Vasquez, M. E. Bastin, B. T. Baune, J. C. Beckham, J. Blangero, D. I. Boomsma, H. Brodaty, H. G. Brunner, R. L. Buckner, J. K. Buitelaar, J. R. Bustillo, W. Cahn, M. J. Cairns, V. Calhoun, V. J. Carr, X. Caseras, S. Caspers, G. L. Cavalleri, F. Cendes, A. Corvin, B. Crespo-Facorro, J. C. Dalrymple-Alford, U. Dannlowski, E. J. C. de Geus, I. J. Deary, N. Delanty, C. Depondt, S. Desrivières, G. Donohoe, T. Espeseth, G. Fernández, S. E. Fisher, H. Flor, A. J. Forstner, C. Francks, B. Franke, D. C. Glahn, R. L. Gollub, H. J. Grabe, O. Gruber, A. K. Håberg, A. R. Hariri, C. A. Hartman, R. Hashimoto, A. Heinz, F. A.

Henskens, M. H. J. Hillegers, P. J. Hoekstra, A. J. Holmes, L. E. Hong, W. D. Hopkins, H. E. H. Pol, T. L. Jernigan, E. G. Jönsson, R. S. Kahn, M. A. Kennedy, T. T. J. Kircher, P. Kochunov, J. B. J. Kwok, S. L. Hellard, C. M. Loughland, N. G. Martin, J.-L. Martinot, C. M. Donald, K. L. McMahon, A. Meyer-Lindenberg, P. T. Michie, R. A. Morey, B. Mowry, L. Nyberg, J. Oosterlaan, R. A. Ophoff, C. Pantelis, T. Paus, Z. Pausova, B. W. J. H. Penninx, T. J. C. Polderman, D. Posthuma, M. Rietschel, J. L. Roffman, L. M. Rowland, P. S. Sachdev, P. G. Sämann, U. Schall, G. Schumann, R. J. Scott, K. Sim, S. M. Sisodiya, J. W. Smoller, I. E. Sommer, B. S. Pourcain, D. J. Stein, A. W. Toga, J. N. Trollor, N. J. A. Van der Wee, D. van ‘t Ent, H. Völzke, H. Walter, B. Weber, D. R. Weinberger, M. J. Wright, J. Zhou, J. L. Stein, P. M. Thompson, S. E. Medland; Enhancing Neuro Imaging Genetics through Meta-Analysis Consortium (ENIGMA)—Genetics working group, The genetic architecture of the human cerebral cortex. *Science* **367**, eaay6690 (2020).

57. A. F. Alexander-Bloch, A. Raznahan, S. N. Vandekar, J. Seidlitz, Z. Lu, S. R. Mathias, E. Knowles, J. Mollon, A. Rodrigue, J. E. Curran, H. H. H. Görring, T. D. Satterthwaite, R. E. Gur, D. S. Bassett, G. D. Hoftman, G. Pearlson, R. T. Shinohara, S. Liu, P. T. Fox, L. Almasy, J. Blangero, D. C. Glahn, Imaging local genetic influences on cortical folding. *Proc. Natl. Acad. Sci. U.S.A.* **117**, 7430–7436 (2020).

58. M. F. Glasser, S. N. Sotiropoulos, J. A. Wilson, T. S. Coalson, B. Fischl, J. L. Andersson, J. Xu, S. Jbabdi, M. Webster, J. R. Polimeni, D. C. Van Essen, M. Jenkinson; WU-Minn HCP Consortium, The minimal preprocessing pipelines for the Human Connectome Project. *Neuroimage* **80**, 105–124 (2013).

59. D. C. Van Essen, S. M. Smith, D. M. Barch, T. E. J. Behrens, E. Yacoub, K. Ugurbil; WU-Minn HCP Consortium, The WU-Minn Human Connectome Project: An overview. *Neuroimage* **80**, 62–79 (2013).

60. S. Kharabian Masouleh, S. B. Eickhoff, Y. Zeighami, L. B. Lewis, R. Dahnke, C. Gaser, F. Chouinard-Decorte, C. Lepage, L. H. Scholtens, F. Hoffstaedter, D. C. Glahn, J. Blangero, A. C. Evans, S. Genon, S. L. Valk, Influence of Processing Pipeline on Cortical Thickness Measurement. *Cereb. Cortex* **30**, 5014–5027 (2020).

61. J. E. Schmitt, G. L. Wallace, M. A. Rosenthal, E. A. Molloy, S. Ordaz, R. Lenroot, L. S. Clasen, J. D. Blumenthal, K. S. Kendler, M. C. Neale, J. N. Giedd, A multivariate analysis of neuroanatomic relationships in a genetically informative pediatric sample. *Neuroimage* **35**, 70–82 (2007).

62. L. Almasy, J. Blangero, Multipoint quantitative-trait linkage analysis in general pedigrees. *Am. J. Hum. Genet.* **62**, 1198–1211 (1998).

63. P. Kochunov, B. Patel, H. Ganjgahi, B. Donohue, M. Ryan, E. L. Hong, X. Chen, B. Adhikari, N. Jahanshad, P. M. Thompson, D. Van't Ent, A. den Braber, E. J. C. de Geus, R. M. Brouwer, D. I. Boomsma, H. E. Hulshoff Pol, G. I. de Zubicaray, K. L. McMahon, N. G. Martin, M. J. Wright, T. E. Nichols, Homogenizing Estimates of Heritability Among SOLAR-Eclipse, OpenMx, APACE, and FPHI Software Packages in Neuroimaging Data. *Front. Neuroinform.* **13**, 16 (2019).

64. L. Almasy, T. D. Dyer, J. Blangero, Bivariate quantitative trait linkage analysis: Pleiotropy versus co-incident linkages. *Genet. Epidemiol.* **14**, 953–958 (1997).

65. D. Zheng, J. Chen, X. Wang, Y. Zhou, Genetic contribution to the phenotypic correlation between trait impulsivity and resting-state functional connectivity of the amygdala and its subregions. *Neuroimage* **201**, 115997 (2019).

66. L. D. Griffin, The intrinsic geometry of the cerebral cortex. *J. Theor. Biol.* **166**, 261–273 (1994).

67. M. P. Noonan, M. E. Walton, T. E. J. Behrens, J. Sallet, M. J. Buckley, M. F. S. Rushworth, Separate value comparison and learning mechanisms in macaque medial and lateral orbitofrontal cortex. *Proc. Natl. Acad. Sci. U.S.A.* **107**, 20547–20552 (2010).

68. M. P. Noonan, J. Sallet, R. B. Mars, F. X. Neubert, J. X. O'Reilly, J. L. Andersson, A. S. Mitchell, A. H. Bell, K. L. Miller, M. F. S. Rushworth, A neural circuit covarying with social hierarchy in macaques. *PLOS Biol.* **12**, e1001940 (2014).

69. M. G. Baxter, A. C. Santistevan, E. Bliss-Moreau, J. H. Morrison, Timing of cyclic estradiol treatment differentially affects cognition in aged female rhesus monkeys. *Behav. Neurosci.* **132**, 213–223 (2018).

70. T. Rinne, R. S. Muers, E. Salo, H. Slater, C. I. Petkov, Functional imaging of audio-visual selective attention in monkeys and humans: How do lapses in monkey performance affect cross-species correspondences? *Cereb. Cortex* **27**, 3471–3484 (2017).

71. X.-N. Zuo, X.-X. Xing, Effects of non-local diffusion on structural MRI preprocessing and default network mapping: Statistical comparisons with isotropic/anisotropic diffusion. *PLOS ONE* **6**, e26703 (2011).

72. P. A. Yushkevich, J. Piven, H. C. Hazlett, R. G. Smith, S. Ho, J. C. Gee, G. Gerig, User-guided 3D active contour segmentation of anatomical structures: Significantly improved efficiency and reliability. *Neuroimage* **31**, 1116–1128 (2006).

73. B. Fischl, FreeSurfer. *NeuroImage* **62**, 774–781 (2013).

74. C. J. Donahue, S. N. Sotiropoulos, S. Jbabdi, M. Hernandez-Fernandez, T. E. Behrens, T. B. Dyrby, T. Coalson, H. Kennedy, K. Knoblauch, D. C. Van Essen, M. F. Glasser, Using diffusion tractography to predict cortical connection strength and distance: A quantitative comparison with tracers in the monkey. *J. Neurosci.* **36**, 6758–6770 (2016).

75. K. B. Nooner, S. J. Colcombe, R. H. Tobe, M. Mennes, M. M. Benedict, A. L. Moreno, L. J. Panek, S. Brown, S. T. Zavitz, Q. Li, S. Sikka, D. Gutman, S. Bangaru, R. T. Schlachter, S. M. Kamiel, A. R. Anwar, C. M. Hinz, M. S. Kaplan, A. B. Rachlin, S. Adelsberg, B. Cheung, R. Khanuja, C. Yan, C. C. Craddock, V. Calhoun, W. Courtney, M. King, D. Wood, C. L. Cox, A. M. C. Kelly, A. D. Martino, E. Petkova, P. T. Reiss, N. Duan, D. Thomsen, B. Biswal, B. Coffey, M. J. Hoptman, D. C. Javitt, N. Pomara, J. J. Sidtis, H. S. Koplewicz, F. X. Castellanos, B. L. Leventhal, M. P. Milham, The NKI-rockland sample: A model for accelerating the pace of discovery science in psychiatry. *Front. Neurosci.* **6**, 152 (2012).

76. P. Kochunov, B. Donohue, B. D. Mitchell, H. Ganjgahi, B. Adhikari, M. Ryan, S. E. Medland, N. Jahanshad, P. M. Thompson, J. Blangero, E. Fieremans, D. S. Novikov, D. Marcus, D. C. Van Essen, D. C. Glahn, L. E. Hong, T. E. Nichols, Genomic kinship construction to enhance genetic analyses in the human connectome project data. *Hum. Brain Mapp.* **40**, 1677–1688 (2019).

77. M.-M. Mesulam, *Principles of Behavioral and Cognitive Neurology* (Oxford University Press, 2000), pp. 1–120.

78. T. Yarkoni, R. A. Poldrack, T. E. Nichols, D. C. Van Essen, T. D. Wager, Large-scale automated synthesis of human functional neuroimaging data. *Nat. Methods* **8**, 665–670 (2011).
